# Curing piglets from diarrhea and preparation of a healthy microbiome with *Bacillus* treatment for industrial animal breeding

**DOI:** 10.1038/s41598-020-75207-1

**Published:** 2020-11-10

**Authors:** Shousong Yue, Zhentian Li, Fuli Hu, Jean-François Picimbon

**Affiliations:** 1 Biotechnology Research Center, Shandong Academy of Agricultural Sciences, Jinan, China; 2 College of Animal Sciences and Technology, Henan Agricultural University, Zhengzhou, China; 3 College of Animal Sciences and Technology/College of Veterinary Medicine, Huazhong Agricultural University, Wuhan, China; 4 School of Bioengineering, Qilu University of Technology, Jinan, China

**Keywords:** Diseases, Infectious diseases, Bacterial infection, Clostridium difficile, Microbiology, Clinical microbiology, Microbial communities, Infectious-disease diagnostics, Pathogens

## Abstract

High-throughput farming of animals for an essential purpose such as large scale health and production of hogs is a challenge for the food industry in the modern world. The problem is that the breeding of livestock for fast growth or high yields of meat is often associated with illness and microbial infection that develop under the breeding conditions. Piglet diarrhea is most common pig disease, leading to heavy mortality and thereby economic loss. We proved that chemical drugs can relieve the symptoms of diarrhea in ill piglets, but they do not treat the underlying cause, i.e. significantly altered bacterial gut flora. Using Illumina sequencing of fecal DNA, we showed that the bacterial gut flora of piglets treated with antibiotics remain close to the ill conditions. However, using Illumina sequencing of fecal DNA from piglets treated with a specific *Bacillus* (*Bacillus subtilis* Y-15, *B. amyloliquefaciens* DN6502 and *B. licheniformis* SDZD02) demonstrated the efficiency of natural bioproducts not only on curing diarrhea, but also on beneficial bacteria to re-establish in the piglet gut. We therefore propose a new natural “medicine” to be explored by the world farm animal agriculture industry, particularly for sustainable improvement of swine livestock production and health.

## Introduction

Animal breeding is an old practice developed by humans for the production of dairy foods (cattle), transport (horses), rodeo or recreational events (dogs and bison for instance). The task of domestication and/or specific animal breeding at the industrial level includes controlled mating, successful reproduction of captive species and mass production of selected strains for our own consumption or pleasure. This dates back to the Neolithic period (about 7000 BC) when the men started to settle down and organize food in tribes and villages before cities and urban areas existed.

The task has always been hard and difficult, but it becomes strictly necessary now and then as we have stopped hunting and abandoned our daily milk and meat food supply to depend only on intensive animal farming and/or mass-production. Animal production for human consumption needs to drastically increase worldwide now as the human population grows nowadays at an unprecedented rate. Modern animal production practices lay on sustainable maintenance of health conditions in livestock that largely rely on the (too) extensive use of antimicrobial drugs^1^.

Similarly to many other domesticated animals, i.e. those raised in an agricultural setting to produce food, pigs and piglets suffer various infections that can come from their environment, nutrition, internal parasites, viruses, bacterial microbes and/or a combination of all^2^. This represents a major threat for agronomic health and eventually for human health, as there are cases of influenza or other infectious disease passed on to humans from pigs^3,4^. Therefore, studying microbial diseases in swine may help not only to improve livestock health conditions but also envision new treatment for human infectious diseases^5^. One of the main diseases related to microbes and piglet livestock is known as piglet diarrhea that can have devastating outcomes on animal health and thereby food production industry as most recently documented in China^6^.

Porcine or Piglet Epidemic Diarrhea (PED) can rapidly spread through vomit and feces in livestock, resulting in loss of appetite and severe dehydration if not death. Ill animals usually grow sick and lose weight, which strongly challenges the ethics and the meat supply quality at the international level^7,8^.

Piglet diarrhea or “*scour*”, an excretion of feces containing excess fluid in 5–15 days-old pigs, is usually caused by various strains of *Coccidia* (*Isospora suis*), *Clostridial enteritis*, *Escherichia coli*, *Salmonella choleraesuis* and *Brachyspira hampsonii/hyodysenteriae*, among many other bacterial pathogens that proliferate in poor hygiene, housing and/or feeding conditions^9–14^. Elimination of noxious agents by medication has been early used and as early proved to have various limitations, the most important of them being some adverse effects on the animals and human (hives, breathlessness, swelling, vomiting, seizures, fevers, blood in the urine, bloody diarrhea, etc.)^15^. Use of antibiotics to treat bacterial and parasitic infections may become more dangerous than inoculating the microbe or the virus itself, especially over a long-term medication as shown for amoxicillin^16^. Another problem is the multi-drug resistance capacity that is often developed by specific microbial isolates and hamper treatment^17^. Finally, using antibiotics have been shown to drastically alter animal gut flora, which urged to find new remedies to treat piglet infections following epidemic diarrhea outbreaks^18,19^.

Various non antibiotic strategies including feed additives such as acidifiers, prebiotics, yeast products and/or plant oil chemicals have been proposed in diets for pigs as an alternative to antibiotic molecule^20^. However, in some cases, using plant oils as alternatives to antibiotics was not without side effects in piglets^21^. Therefore, more promising alternatives were suggested from using probiotics such as *Bacillus* strains or *Lactobacillus *sp. to stimulate digestive enzyme activity and gut integrity, and thereby immune system and growth performance in swine^22–26^, similarly to studies of hyperlipidemia rodent models^27–29^.

This study aimed to find a new medicine to be applied in agro-alimentary food industry, in particular in prevention and curing of piglet diarrhea. To achieve this, the tripartite composition of a specific *Bacillus* (*B.*) strain-supplemented formula (*Bacillus subtilis* Y-15, *B. amyloliquefaciens* DN6502 and *B. licheniformis* SDZD02) was produced in a microbiology laboratory industrial platform from Shandong and delivered with food nutrients to piglets in experimental animal farms from Henan Province (China). The recovery of healthy conditions was observed in piglets treated with *Bacillus*, similarly to the group of piglets treated with medicinal chemical drugs (Colistin and Kitasamycin). Then, we collected piglet feces and analyzed their bacterial composition by using Illumina DNA sequencing. The overall result of diarrhea treatment with *Bacillus* formula was beneficial to an extent not reported before. In contrast to Colistin and Kitasamysin that had a marked negative effect on gut flora, applying a microecosystem such as *Bacillus* not only cured diarrhea, but also restored healthy gut flora in livestock.

## Results

### Overall comparison of microbiomes in piglet groups in relation with infectious diarrhea and curing

**Figure 1:**
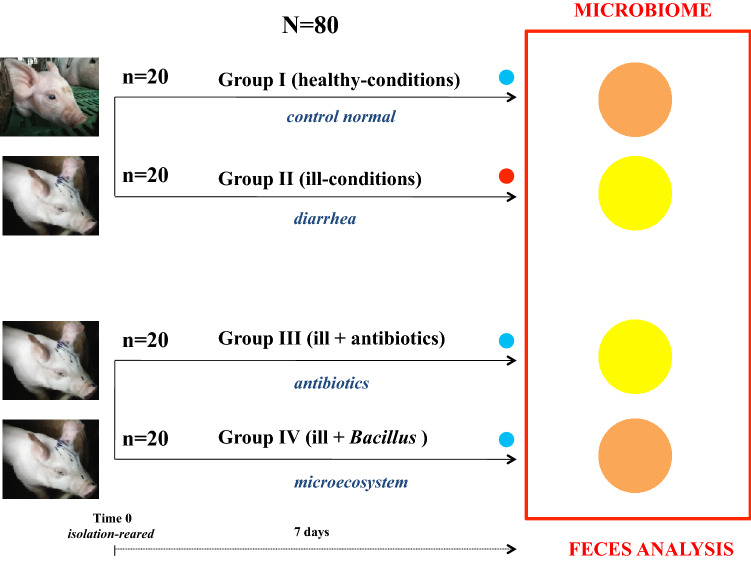
Experimental model of testing *Bacillus* and antibiotics on piglet diarrhea in industrial farming conditions. The red dot shows the groups that still suffer infectious diarrhea symptoms after 7 days. The blue dot shows the groups that have recovered from infectious diarrhea or show healthy conditions. Time 0 is when diarrhea symptoms begin in ill-groups. Feces were collected in each group after 7 days treatment. The circle in orange shows healthy microbiomes. The circle in yellow shows ill microbiomes. N = total number of piglets tested, n = number of piglets per group.

**Figure 2:**
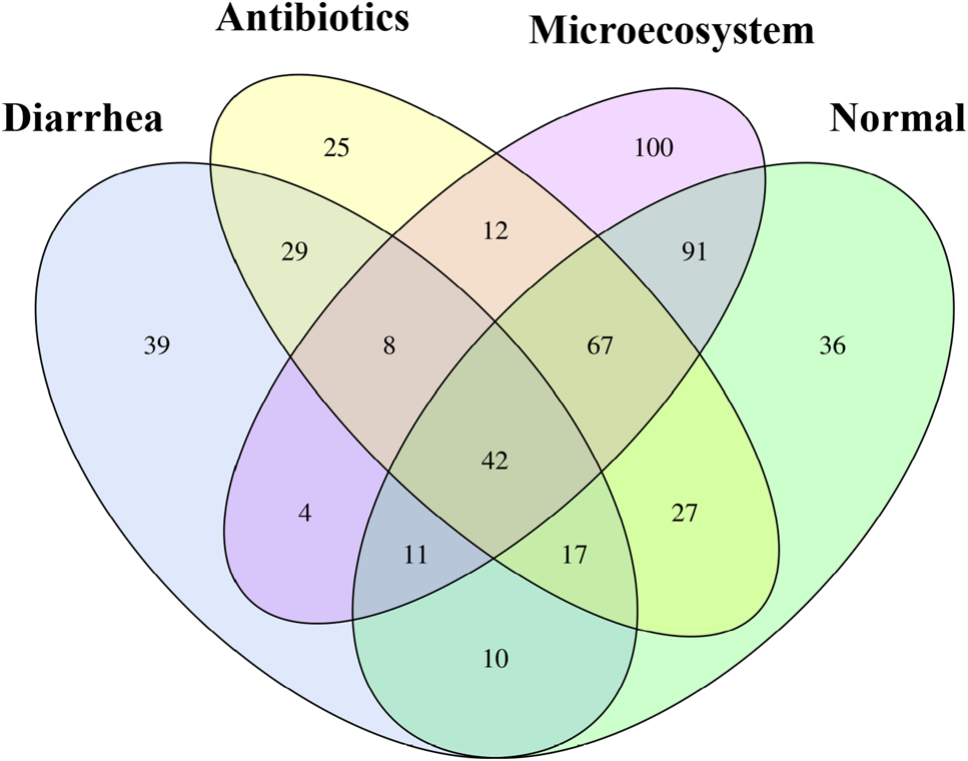
Venn diagram of shared OTUs across different piglet groups in relation with ill-diarrhea and curing. Different colors represent different groups: (1) normal, (2) ill-diarrhea, (3) antibiotics and (4) microecosystem. The interior of each circle symbolically represents the number of observed OTUs in each sample or group. The overlapping area or intersection represents the set of OTUs commonly present in the counterpart samples or groups (diarrhea/antibiotics; microecosystem/normal). The single-layer zone represents the number of OTUs uniquely found in specific sample/group (VenDiagram, R(v3.1.1), BGI Co., Ltd).

**Figure 3:**
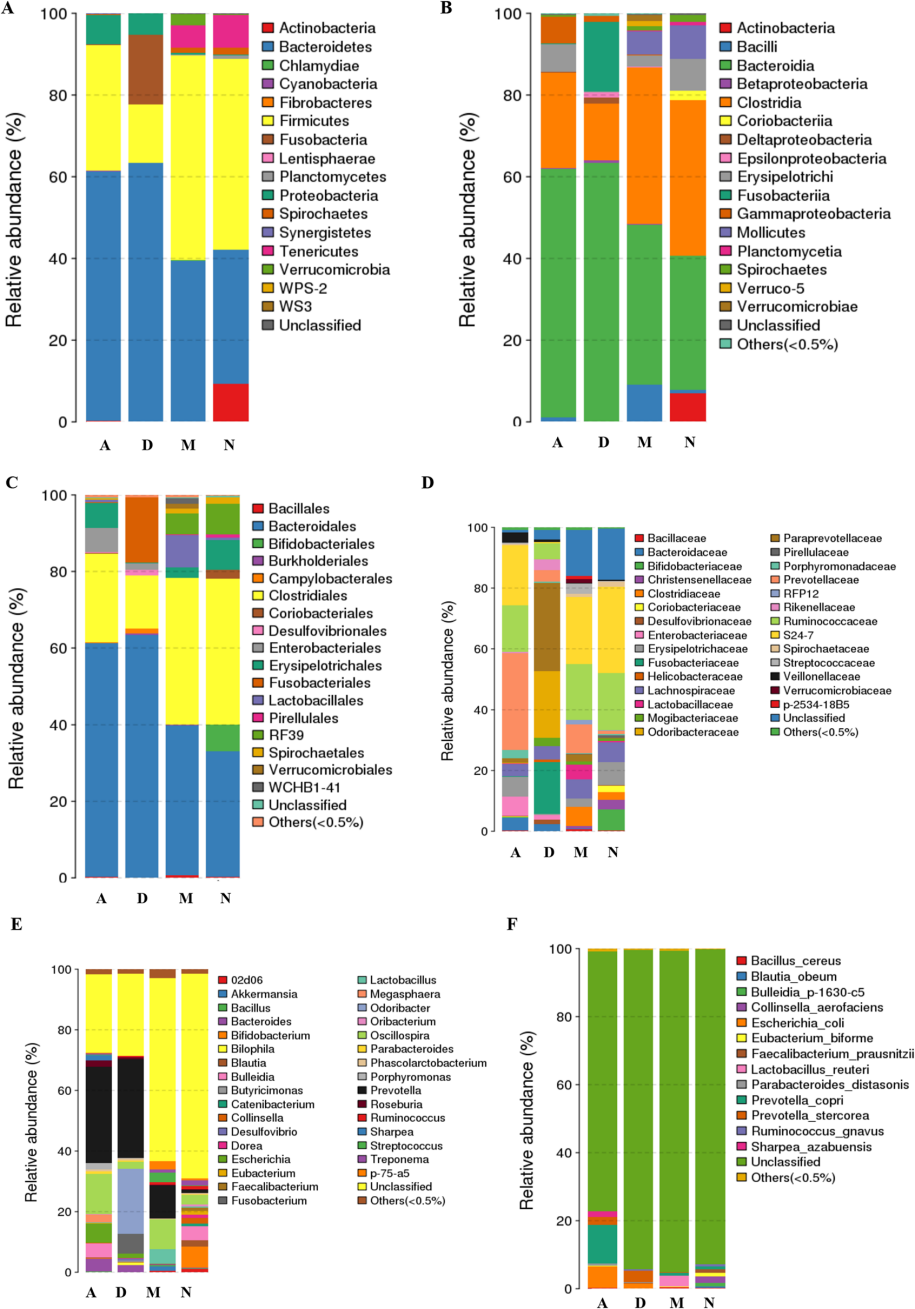
Taxonomic composition distribution in piglet samples related to ill-diarrhea and curing at phylum, order, class, family, genus and species level. (**A**) Phylum-level. (**B**) Class-level, (**C**) Order-level, (**D**) Family-level, (**E**) Genus-level and (**F**) Species-level. The ratio of each species in a specific sample is directly displayed (color code). At phylum-level, all species were used to draw the histogram. The species of which abundance is less than 0.5% in all samples were classified into “others” in other ranks (ade4, R(v3.1.1), BGI Co., Ltd).

**Figure 4:**
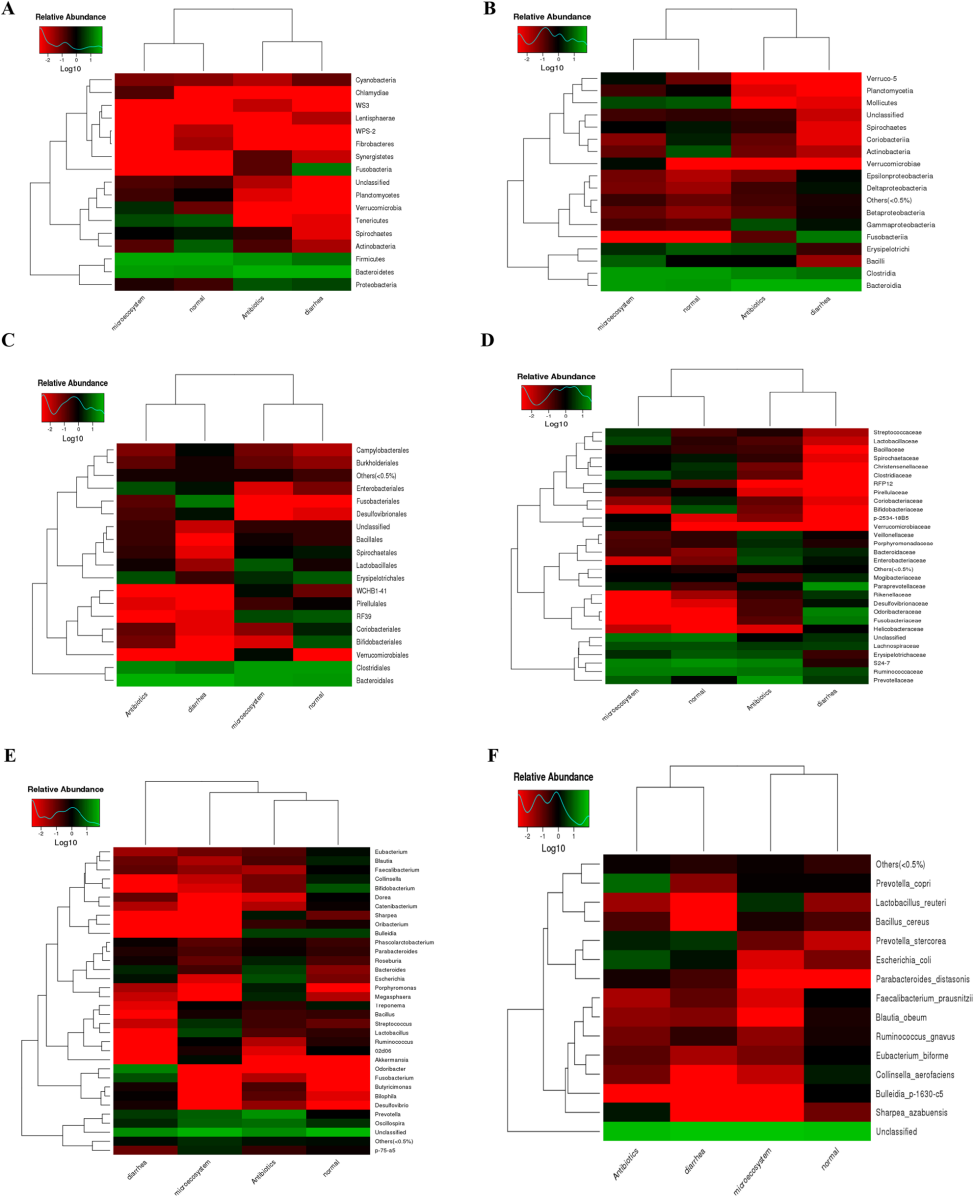
Bacterial species clustering based on the abundance of each species (heat map) at different taxonomic rank in piglets samples in relation with ill-diarrhea and curing. Log-scaled percentage heat map of phylum- (**A**), class- (**B**), order- (**C**), family- (**D**), genus- (**E**) and species- (**F**) level. Longitudinal clustering indicates the similarity of all species among the four piglet samples. The horizontal clustering indicates the similarity of certain species among the four samples. The closer the distance is and the shorter the branch length is, the more similar the species composition is between the samples. At phylum, all species were used to draw the heatmap. The species of which abundance is less than 0.5% in all samples were classified into “others” in other ranks. The individual bacterial species are represented as colors (gplots, R(v3.1.1), BGI Co., Ltd).

**Figure 5:**
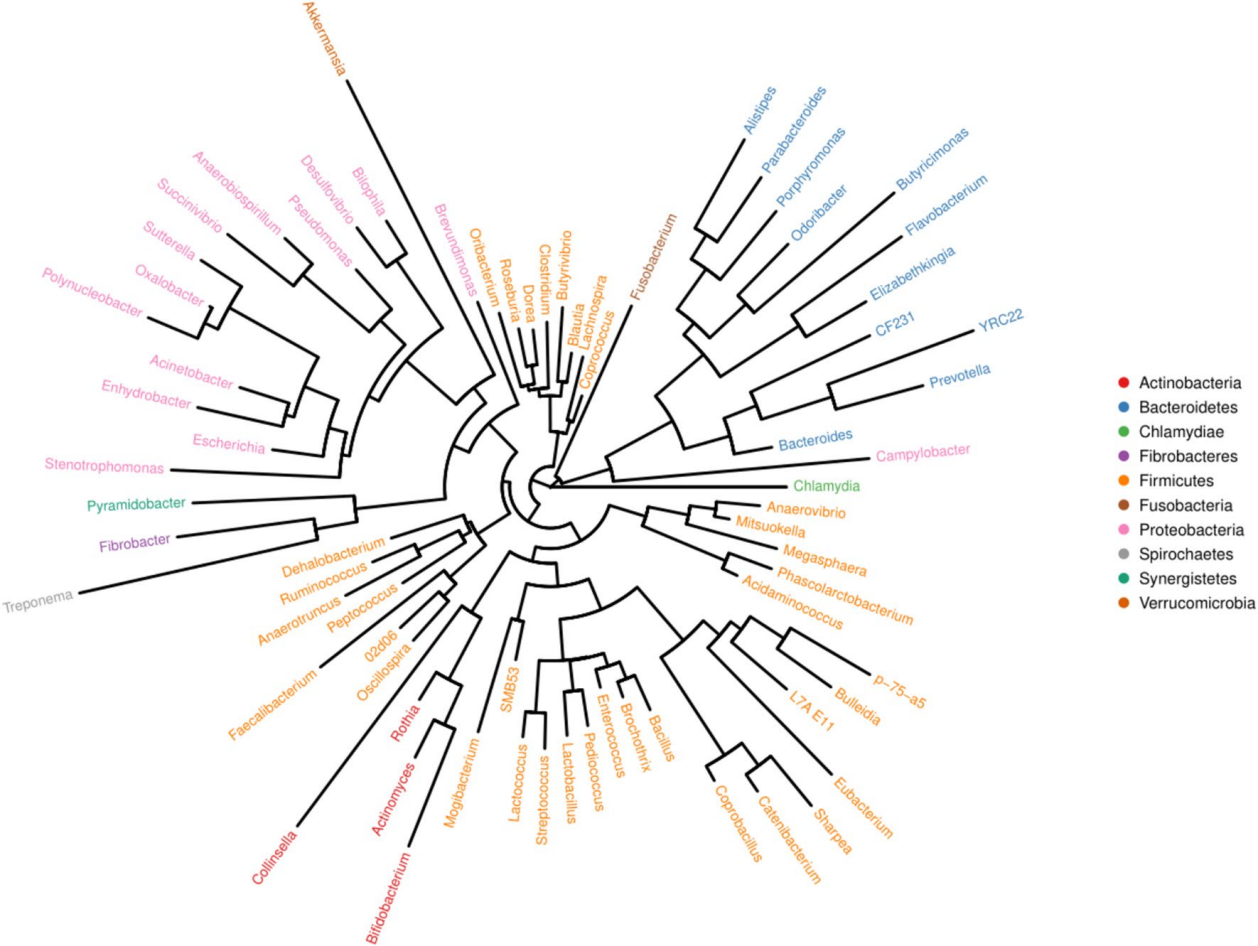
Genus level phylogenetic tree. The same phylum is shown as the same color (PyNAST, QIIME, R(v3.1.1), BGI Co. Ltd).

Our study explores the fecal microbiota of piglets after being challenged with a cocktail of probiotic *Bacillus* spp. The topic is of significance, as pigs used for livestock are commonly infected with diarrhea causing ecological loss worldwide. The study design includes four different groups (1) healthy control and infected piglets (diarrhea), which were either (2) untreated, (3) treated with antibiotics (AB) or (4) treated with *Bacillus* spp (Fig. 1). The microbiome was assessed using 16S rRNA gene sequencing using Illumina HiSeq2500 platform (Figs. 2, 3, 4, 5 and Figures S1–S3).

The pictorial representation of the relationships between the four groups (Venn Diagram) in relation with piglet diarrhea and curing showed a strong relation between healthy control and microecosystem (Fig. 2). In contrast, the position, the configuration and overlap of the circles indicating the relationships between the groups clearly showed healthy control/microecosystem and ill-diarrhea/AB association, respectively (Fig. 2). Therefore, it clearly showed that the core of microbiomes in the microecosystem and healthy groups was very closely related, much more than with that of the AB group (Fig. 2).

The number of Operational Taxonomic Units (OTUs) in a sample primarily represents the degree of sample diversity. OTU numbers were higher in the microsystem group (350), much higher than the diarrhea and AB group (only 160 and 227, respectively, see Table 1). Based on OTU numbers, the degree of microecosystem sample diversity was close to healthy control sample diversity (335 vs 301, Table 1). The differences of OTU composition in the four groups were also displayed by principal component analysis (PCA) and OTU rank curve (Figure S1). PCA showed similarity of the microecosystem and healthy control samples at overall community level (Figure S1A). Similarly, OTU rank-abundance distribution curves representing species richness and species evenness showed more bacterial diversity occurring in microecosystem and healthy control groups compared to AB and ill-diarrhea groups (Figure S1B).

**Table 1:** Operational taxonomic unit (OTU) statistics (97% threshold).

Sample	Tag number	OTU number
Antibiotics	18,549	227
Diarrhea	19,477	160
Microecosystem	16,848	335
Normal	17,942	301

OTU number per sample primarily represents the degree of sample diversity.

### Profiling microbial composition in four different groups of piglets in relation with infectious diarrhea and treatment

The taxonomics composition distribution histograms of each sample were shown at phylum, order, class, family, genus, species level separately (Fig. 3; Figure S2). The dominant bacterial phyla (relative abundance ≥ 1%) varied with health conditions and/or specific treatments (Figure S2). Ill-diarrhea group had about 63.32% Bacteroidetes, 17.04% Fusobacteria and 14.27% Firmicutes, similarly to AB group (61.19% Bacteroidetes, 30.91% Firmicutes and 7.08% Proteobacteria; Figure S2). Control healthy and microecosystem groups had higher percent Firmicutes (46.73–50.10%) and lower percent Bacteroidetes (32.77–39.27%), in addition to the Tenericutes (5.62–8.06%) and the Spirochaetes (1.10–1.68%). Microecosystem group also had 2.72% Verrucomicrobia, while healthy control group had more percent Actinobacteria (9.27%; Figure S2).

Comparative analysis between healthy and ill samples showed ill-diarrhea very significantly associated with decreased abundance of Firmicutes and Actinobacteria (p < 0.01), as well as with increased abundance of Bacteroidetes and Fusobacteria (p < 0.01). Similarly, comparative analysis between AB and microecosystem samples showed antibiotics very significantly associated with decreased abundance of Firmicutes (p < 0.05), Tenericutes (p < 0.01) and Verrucomicrobia (p < 0.05), as well as with significantly increased abundance of Bacteroidetes (p < 0.05) and Proteobacteria (p < 0.01; Figure S2).

The most abundant microbial phyla of healthy control piglets were Firmicutes, Bacteroidetes, Tenericutes, Actinobacteria, Fibrobacteres and Synergistetes. Except Actinobacteria, all the phyla (Firmicutes, Bacteroidetes, Tenericutes, Fibrobacteres and Synergistetes) were retrieved at normal levels in microecosystem samples, not in AB samples (Fig. 3A). In addition, the profiling of microbial phyla in ill-diarrhea piglets was characterized by a ratio low Firmicutes/high Bacteroidetes, high fusobacteria and high proteobacteria, which is probably a specific profiling for diagnostic of diarrhea disease in piglets (Fig. 3A). Importantly, the ill-profiling was not significantly changed by the antibiotic treatment. Antibiotics only removed Fusobacteria, but neither Proteobacteria level nor the ratio of Firmicutes and Bacteroidetes showed any changes with drug treatment (Fig. 3A). The most abundant microbial class were Clostridia, Bacteroidia, Mollicutes, Erysipelotrichia and Actinobacteria, which was a bacterial class profiling very close to that observed in microecosystem but not in AB samples (Fig. 3B). Antibiotics did not improve mollicutes level. It stimulated Gammaproteobacteria level instead (Fig. 3B). Diarrhea was associated with high levels of Fusobacteria, Gammaproteobacteria, Epsilonproteobacteria, Betaproteobacteria, and reduction of Clostridia/ increase of Bacteroidia (Fig. 3B). The levels of Clostridia remained low, while Bacteroidia levels remained high in piglets treated with AB (Fig. 3B). Bacteroidales, Clostridiales, RF39, Bifidobacteriales, Spirochaetales, Coriobacteriales, and Pirellulales were the most abundant microbial orders in healthy control piglet samples (Fig. 3C). Bacillus treatment stimulated microbial order diversity, with increasing levels of Verrucomicrobiales, WCHB1-41 and Lactobacillales. The levels of Clostridiales and Bacteriodales were maintained in microecosystem-treated samples (Fig. 3C). In contrast, low Clostridiales/high Bacteriodales levels were characteristic of diarrhea conditions, which was not improved by antibiotic treatments (Fig. 3C). Fusobacteriales, enterobacteriales, desulfovibrionales and campylobacterales were numerous in piglet diarrhea conditions (Fig. 3C). The levels of bacterial orders such as enterobacteriales remained high after antibiotic treatments (Fig. 3C). The main microbial families in healthy control and bacillus-treated piglet samples were *Bacteroidaceae*, *S24-7*, *Ruminococcaceae *and *Prevotellaceae*, while ill piglets showed high levels of *Desulfovibrionaceae*, *Odoribacteraceae* and *Fusobacteriaceae* (Fig. 3D). Both antibiotics and bacillus-treatments increased the levels of bacteria such as *Prevotellaceae*, *S24-7* and *Ruminococcaceae*, but the *Bacteriodaceae* levels remained low in AB samples, similar to that of ill-conditions (Fig. 3D). Other microbe families such as *Enterobacteriaceae* and *Bacteroidaceae* were found in both ill and AB samples, showing that antibiotic treatments did not restore healthy gut flora (Fig. 3D). Piglets with clinical signs of diarrhea (ill) and the AB group harbored different compositions of the bacterial profiles from healthy control and bacillus-treated piglets also on the basis of taxonomic composition distribution of genus-level (Fig. 3E). *Prevotella*, *Odoribacter*, *Fusobacterium*, *Escherichia* and *Treponema* were predominant in ill pigs. *Prevotella*, *Escherichia* and *Treponema* levels remained high after antibiotic treatments (Fig. 3E). The overall microbial diversity at the genus-level was much higher in normal and bacillus-treated piglets. In particular, high levels of *Bilophila* were found to be associated to healthy control and *Bacillus* conditions (Fig. 3E). Interestingly, major changes in bacterial species composition were also observed in feces from diarrhea-ill piglets. Species diversity was rather low in ill-animals. Piglets suffering infectious diarrhea had high levels of *Ruminococcus gnavus*, *Prevotella stercorea* and *E. coli* (Fig. 3F). Much more diversity was found in healthy control normal conditions characterized by various microbial species such as *Faecalibacterium prausnitzii, Eubacterium biforme, Prevotella copri* and *Collinsella aerofaciens.* Even more interestingly, the species diversity was not as high in both antibiotics and bacillus treatments. However, microecosystem samples were deprived of *R. gnavus*, *P. stercorea* and *E. coli* bacteria, while AB samples still showed high levels of main toxic bacteria such as *E. coli* and *P. stercorea* (Fig. 3F).

### Inefficient effects of antibiotics on piglet gut flora

The hierarchically clustered heat map analysis associated with the similarity of bacterial flora compositions performed at the phylum, class, order, family and species level disclosed a richness and diversity of bacteria in the feces of antibiotics-treated piglets very close to those observed in the feces of diarrhea piglets. Only heat map based on genus-level grouped AB with microecosystem and healthy control conditions (Fig. 4). Importantly, in heat maps (point density maps), like in OTUs and taxonomics histograms, there was a strong correlation between microecosystem/healthy control and AB/diarrhea at the phylum level (Fig. 4A). For many phyla, the overall microbiota proportion did not significantly change throughout the four groups, showing an unchanged baseline in the piglet microbiome. However, some changes to a significantly high degree were observed in some specific phyla that were modulated either by illness and/or treatment (Fig. 4A; Figure S2).

Fusobacteria was a dominant phylum in ill untreated-piglet samples. Fusobacteria abundance in piglet feces was decreased after drug treatments. The levels of Fusobacteria were even lower in feces from piglets treated with *Bacillus*; they reached the levels observed in healthy control piglets (Fig. 4A; Figure S2). Relative abundance of Chlamydiae, WPS-2, Fibrobacteres, Verrucomicrobia and Bacteroidetes was similar between diarrhea and AB (Fig. 4A; Figure S2). Relative abundance of Firmicutes/Bacteroidetes, Spirochaetes, Fusobacteria, Synergistetes, Lentisphaerae, WS3 and Cyanobacteria was similar between healthy and microecosystem (Fig. 4A; Figure S2).

The heat map data of class, order and family abundance also showed a correlation between diarrhea/AB and microecosystem/healthy control (Fig. 4B–D). Bacteroidia, Mollicutes, Verrucomicrobiae and Verruco-5 mainly helped group AB with diarrhea, while Spirochaetes, Fusobacteria and Clostridia helped group microecosystem with normal gut function (Fig. 4B). Heat map of relative abundance at the order-level extracted low Fusobacteriales, high RF39, Clostridiales and Bacteroidales in microecosystem/healthy control group, and low WCHB1-41, Verrucomicrobiales and Bacteroidales in AB/ill group (Fig. 4C). Bacterial family divergence, as shown as a heat map of relative abundance, showed a common profiling between diarrhea and AB in the low microbial density of *RFP12* and *Pirellulaceae*, and in the high microbial density of *Veillonellaceae*, *Fusobacteriaceae* and related bacterial families (Fig. 4D). Microecosystem and healthy control groups were associated with high microbial density of *RFP12* and *Pirellulaceae*, among many others, and low microbial density of *Fusobacteriaceae* and other related bacterial families (Fig. 4D).

### Identification of beneficial bacteria associated with healthy and bioproduct conditions

Log-scaled percentage heat map of species-level in samples related to piglet diarrhea and curing showed that the genera (34 clades) were distributed into five groups or clusters: *Eubacterium*, *Bacteroides*/*Escherichia*, *Bacillus*, *Fusobacterium* and *Prevotella* (Fig. 4E). *Eubacterium* was abundant in healthy control conditions, while *Fusobacterium* and *Prevotella* diagnosed illness (Fig. 4E). Low levels in *Eubacterium* and *Bacillus* groups were also indicative of diarrhea conditions (Fig. 4E). Meanwhile, the heat map showed that a lot of species in *Fusobacterium* group decreased after probiotics (microecosystem) or AB treatment (Fig. 4E). The most notable decrease not only of *Fusobacterium* but also of *Butyricimonas*, *Bilophila* and *Desulfovibrio* was found in *bacillus*-treated piglets (Fig. 4E). In the other groups, the drug or probiotic effect was depending on genus. For instance, in *Eubacterium* group, *Bulleidia* abundance increased during drug treatment, not during *Bacillus* treatment (Fig. 4E). In contrast, abundance of *Treponema*, *Streptococcus*, *Lactobacillus*, *Ruminococcus*, *02d06* and *Akkermansia* in *Bacillus* group increased specifically close to healthy control conditions during microecosystem probiotic treatment (Fig. 4E). In some other cases such as the bacterial genus *p-75-a5*, there were no changes in bacterial abundance in response to AB treatments. However, there were significant abundance changes for healthy control pigs and after *Bacillus* treatment in a bacterial genus such as *p-75-a5* decreased in its abundance during the infection (Fig. 4E).

Finally, the abundance of species with the highest scores in the random piglet sample analysis is represented in a heat map (Fig. 4F). The selected fifteen species segregated into two main groups, *Prevotella copri*/*Parabacteroides distasonis* and *Faecalibacterium prausnitzii*/*Sharpea azabuensis*, according to their relative abundance in samples related to piglet diarrhea and curing. *F. prausnitzii*/*S. azabuensis* cluster exhibited prevalence in healthy control conditions, while *P. stercorea* and *E. coli* in the other cluster were predominant in ill untreated and AB-treated piglets (Fig. 4F). Decrease of bacteria in *P. stercorea* and *E. coli* cluster was associated with *bacillus* treatment and healthy conditions (Fig. 4F). Decrease of bacteria such as *Lactobacillus reuteri* and *Bacillus cereus* in the same cluster associated with development of infectious diarrhea (Fig. 4F). The heat map based on species-level showed high increase of *L. reuteri* and *B. cereus* after probiotic treatment (Fig. 4F). In all, the resulting heat map of species relative abundance showed microecosystem/healthy control and AB/diarrhea clustering, as found for the resulting heat map of class, order and family relative abundance (Fig. 4).

### *Bacillus* bioproduct helps restore healthy gut flora to cure piglet diarrhea

Phylogenetic analysis in the predominant species identified in fecal samples related to piglet diarrhea and curing using the Illumina assay in four groups of piglets (healthy control, microecosystem, AB and ill diarrhea untreated) revealed high density of (1) firmicutes, (2) bacteroidetes, (3) proteobacteria, (4) actinobacteria, and (5) Chlamydiae, Fibrobacteres, Pyramidobacter synergistetes and Treponema (Fig. 5).

Alpha diversity statistics applied for analyzing complexity of species diversity for fecal samples related to piglet diarrhea and curing showed a close similarity mainly between microecosystem and healthy control samples (Table 2). Observed species (sobs), chao1, ace, Shannon and Simpson values all reflected high species richness of bacterial community in microecosystem samples, similarly to control conditions (Table 2). Species richness as measured by sobs, chao1, ace, Shannon and Simpson values all showed higher bacterial diversity than that observed in AB samples (Table 2). Shannon value and Simpson value that reflect the species diversity of the community, affected by both species richness and species evenness, both showed higher abundance of species in microecosystem samples (Table 2).

**Table 2:** Alpha diversity statistics showing the complexity of bacterial diversity for four samples related to piglet diarrhea and curing.

Sample	S obs	Chao1	ACE	H	D
*Antibiotics*	227.000000	249.500000	249.483815	3.559278	0.050575
*Diarrhea*	160.000000	172.000000	177.367134	2.949736	0.113369
*Microecosystem*	335.000000	343.125000	347.306642	4.147630	0.036259
*Normal*	301.000000	331.027778	331.230766	3.701425	0.087531

Alpha-diversity indices Sobs (total number of species observed in sample), Chao1 and ACE (Abundance-based Coverage Estimator) reflect the species richness of community; Shannon’index (H) and Simpson’s index (D) reflect the species diversity of the community.

The complexity of sample is proportional with the first four indices, while with a negative correlation with Simpson’s index.

Species richness and species diversity are highest in microecosystem sample.

Not only the various diversity indexes but also all the rarefaction curves of fecal samples related to piglet diarrhea and curing showed very close similarity between microecosystem and healthy control samples (Figure S3). Over 20,000 sequences, the rarefaction curve between control and microecosystem was similar for observed species, chao, ace and Shannon measurement (Figure S3). The rarefaction of samples based on Simpson value showed a similar curve between microecosystem and AB (Figure S3).

In the last analysis in order to compare the piglet gut microbiota, the groups were classified according to A (healthy and microecosystem) and B (ill and antibiotics). MicrobiomeAnalyst toolkits^30^ were used to analyze alpha- and beta-diversity in A and B. About alpha-diversity (mean species diversity), Chao1 and ACE indices were much greater in group A compared to group B (Figure S4), showing high species richness microbiota in healthy and microecosystem-treated groups of piglets. Shannon and Simpson indices were also much greater in A compared to B (Figure S4), showing that the microbial communities or compositions in healthy and microecosystem-treated piglets are much more diverse than those of piglets suffering from diarrhea or ill-treated with antibiotics. About beta-diversity (change in diversity of species), NonMetric Multidimensional Scaling (NMDS) ordination analysis with Bray–Curtis distance showed that the gut microbiota of group A clearly separates from that of group B (diarrhea and antibiotics groups; Figure S5). The measure distance or distribution value between healthy and microecosystem-treated piglets is very close (Figure S5). This shows that there is very high overall abundance at group A, i.e. that there is a particularly high level of similarity in the gut microbiomes of healthy and microecosystem-treated piglets (Figures S4 and S5).

## Discussion

Piglet diarrhea (or scour) disease common to both the neonatal and the post-weaning stages can be a cause of the high level of mortality in the nursery beds and can seriously challenge the outcome of intensive animal farming or industrial livestock production worldwide. Neonatal diarrhea (ND) and post-weaning diarrhea (PWD) spread unhealthy conditions in swine with the development of enterotoxigenic bacteria such as *E. coli* highly resistant to antibiotic or chemical drugs in the piglet gut^31^. Here, we have analyzed the microbiome of several groups of piglets in relation with diarrhea, revealing the complete infectious microbiome of ill piglets. We show that antibiotic treatments are inefficient to restore the normal healthy control gut bacterial flora in the piglet intestine and therefore unable to re-establish healthy conditions in piglets. As an alternative to drug or antibiotic treatment for swine health, we have performed a comprehensive microbiological medical/industrial study of the impact of three *Bacillus* species not only on piglet diarrhea, but also on gut flora. We have analyzed the piglet response to a new bioproduct. We have demonstrated that the association of *Bacillus subtilis* Y-15, *B. amyloliquefaciens* DN6502 and *B. licheniformis* SDZD02 stimulates beneficial gut flora to cure piglet diarrhea. Here, we present data for the variations in the microbiome of piglet feces under four different conditions: healthy normal, ill diarrhea, drug and microecosystem, respectively, with special attention for bacterial phylum, class, order, family, genus and species associated with piglet diarrhea and its treatment by a new natural biomedical product, *Y15-DN6502-SDZD02*.

There are approximately about 100 species of *bacillus* bacterium. Some can be harmful, but many are safe and extremely beneficial for a natural part of a healthy gut environment when used in mixture with other *Bacillus* strains or healthy bacteria^32,33^. *B. subtilis* has been the subject of probiotic studies to treat many digestive tract problems since the mid-1900s, particularly to treat a variety of maladies which cause diarrhea in infants, young children and elder adults via a stimulatory effect on the immune system^34^. Such effects on the immune system has even brought the attention of using *B. subtilis* for vaccination to enhance immunization programs in developing countries^35^. *B. amyloliquefaciens* is another bacterium of soil origin particularly studied not only for human health, but also for the health of agricultural crops. Many strains of *B. amyloliquefaciens* have been shown to have a very beneficial impact on crops by promoting plant growth, controlling plant disease, or both^36–38^. Similarly, *B. licheniformis* strains have been consumed by humans for better gut health to boost immune system for millions years. Similarly to *B. subtilis*, it can resist to extreme heat but has the property to produce multiple enzymes (proteases, alpha-amylases, beta-mannanases and pectinolytic enzymes) particularly efficient to prevent the growth of harmful bacteria or fungi not only in the animal intestine but also in the environment as a contributor to nutrient cycling^39,40^. So, we combined three soil organisms that have been described as human immune regulators, plant growth promoters and/or producers of antimicrobial compounds in an attempt to treat the diarrhea developed by piglets reared in industrial conditions. The results presented here are unequivocally in support of the use of our bioproduct, *Y15-DN6502-SDZD02,* in the treatment of piglet diarrhea disease in industrial farming (see Figs. 1, 2, 3, 4, 5, Figures S1, S2,S3, S4, S5; Tables S1, S2, Tables 1, 2, 3, 4).

The Illumina sequencing profiles correspond to microbiomes from healthy, ill, drug- and bioproduct-treated groups of piglets. They show a significant variation in the composition of the gut flora and therefore a strong impact of the gut flora on piglet health conditions as described for human gut mucosa and pathology^41–43^. Our four groups-level study on the link between gut flora and piglet diarrhea identifies specific gut bacteria linked to illness and provides evidence that a wide range of other bacteria induced by *Bacillus* treatment, not by antibiotics, can restore healthy conditions in piglets reared for industrial purposes. Like in all healthy soils, richness of microbiota species characterizes the gut intestinal microbiome in healthy groups or individuals. Conversely, it is well known that a loss in species diversity is often associated to disease or severe alteration of health conditions in plant or human organisms^44^. The overall abundance and diversity for most prevalent bacterial organisms in piglet feces in relation with infectious diarrhea and curing are presented in Tables 3, 4. Tables 3 and 4 show the richness of microbiota species that characterizes the gut intestinal microbiome in the healthy control piglet group at the level of phylum, class, order, family, genus and species. The group of healthy piglets is shown to retain high levels of Firmicutes, Clostridia, Clostridiales, *Ruminococcaceae*, *S24-7*, *Bifidobacterium* and many various species from *Bacillus cereus* and *Eubacterium biforme* to *Prevotella copri* (see Figs. 3, 4, 5; Tables 3, 4). In contrast, the group of ill piglets (with diarrhea symptoms) is characterized by an increase in the levels of Bacteroidetes, Bacteroidia, Bacteroidales, *Odoribacteraceae*, *Paraprevotellaceae*, *Fusobacterium*, *Odoribacter*, *Prevotella*, *Escherichia coli* and *Prevotella stercorea* (see Figs. 3, 4, 5; Tables 3, 4). Therefore, our Illumina sequencing results show that the physiological status in piglets is dependent upon the frequency of Firmicutes and Bacteroidetes in the gut microbiota, as found in mice and humans. Studies in mice and humans have associated the Firmicutes with weight gain, lipid metabolism-related disease and obesity, and the Bacteroidetes with weight loss and good or improved general physiological conditions^45–48^. Our study in swine associates the Firmicutes with piglet diarrhea and the Bacteroidetes with healthy conditions (Figs. 3, 4, 5; Tables 3, 4). The Firmicute/Bacteroidete ratio might be therefore one of the key and essential criteria to diagnose diarrhea in industrial animal farming. Among other bacterial species, the levels of *P. copri* versus *P. stercorea*, which could be easily quantified by real-time PCR and/or any technique of gradient gel electrophoresis, might be also important to diagnose healthy versus unhealthy conditions^49^. In our study about the health of piglets in industrial farming, *P. copri* is found to be beneficial (see Figs. 3, 4, 5; Tables 3, 4). *P. copri* helps in the digestion of nutrients and keeps specific bacteria at bay. This could have some negative effects as perhaps in some human arthritis^50^, but the increase of *P. copri* is clearly beneficial for the piglets where it could keep some harmful bacteria such as *E. coli* at bay (see Figs. 3, 4, 5; Tables 3, 4). *P. stercorea* is found to have a negative impact, similarly to human where the increased abundance of *P. stercorea* triggers chronic inflammatory disease^51^.

**Table 3:** Composition of the microbiome (Phylum, Class and Order of bacteria) identified in four samples of fecal DNA related to piglet diarrhea and curing.

Sample	Phylum	Class	Order
**Group I** *Healthy*	Actinobacteria Bacteroidetes **Firmicutes** Planctomycetes Proteobacteria Spirochaetes Verrucomicrobiota	Actinobacteria Bacilli Bacteroidia **Clostridia** Coriobacteriia Erysipelotrichi Mollicutes Planctomycetia Spirochaetes Verruchomicrobiae	Bacillales Bacteroidales Bifidobacteriales **Clostridiales** Coriobacteriales Erysipelotrichales Lactobacillales** Pirellulales RF39 Spirochaetales** Unclassified Others
**Group II** *Microecosystem*	Bacteroidetes **Firmicutes** Proteobacteria Spirochaetes Tenericutes Verrucomicrobiota	Bacilli Bacteroidia Betaproteobacteria **Clostridia** Erysipelotrichi Gammaproteobacteria* Mollicutes Planctomycetia Spirochaetes Verruco-5 Verruchomicrobiae	Bacillales Bacteroidales Burkholderiales **Clostridiales** Erysipelotrichales Lactobacillales** Pirellulales RF39 Spirochaetales** Verrucomicrobialles WCHB1-41 Unclassified Others
**Group III** *Diarrhea*	**Bacteroidetes** Firmicutes Fusobacteria Proteobacteria	**Bacteroidia** Betaproteobacteria Clostridia Deltaproteobacteria Epsilonproteobacteria Fusobacteria	**Bacteroidales** Burkholderiales Campylobacterales Clostridiales Desulfovibrionales Enterobacteriales Erysipelotrichales Fusobacteriales Others
**Group IV** *Antibiotics*	**Bacteroidetes** Firmicutes Proteobacteria	Bacilli **Bacteroidia** Betaproteobacteria Clostridia Deltaproteobacteria Erysipelotrichi Fusobacteria Gammaproteobacteria* Spirochaetes	Bacillales **Bacteroidales** Campylobacterales Clostridiales Coriobacteriales Desulfovibrionales Enterobacteriales Erysipelotrichales Fusobacteriales Lactobacillales** Spirochaetales** Unclassified Others

The most abundant bacteria in the sample or group of piglets are shown in bold. Single asterisk (*) shows bacterial classes increased in both antibiotics and microecosystem groups. Double asterisk (**) shows bacterial orders present in healthy piglets and increased in both antibiotics and microecosystem groups.

**Table 4:** Composition of the microbiome (Family, Genus and Species of bacteria) identified in four samples of fecal DNA related to piglet diarrhea and curing.

Sample	Family	Genus	Species
**Group I** *Healthy*	Bacillaceae** Bifidobacteriaceae Christensenellaceae Clostridiaceae Coriobacteriaceae Desulfovibrionaceae Erysipelotrichaceae Lachnospiraceae Lactobacillaceae** Mogibacteriaceae Paraprevotellaceae Pirellulaceae Porphyromonodaceae Prevotellaceae RFP12 **Ruminococcaceae** **S24-7** Spirochaetaceae** Veillonellaceae Unclassified Others	02d06 Bacillus Bacteroides **Bifidobacterium** Blautia Bulleidia Catenibacterium Collinsella Dorea Eubacterium Faecalibacterium Lactobacillus Oribacterium Oscillospira Parabacteroides Phascolarctobacterium Prevotella Ruminococcus Streptococcus Treponema p75-a5 **Unclassified** Others	*Bacillus cereus* *Blautia obeum* *Bulleidia p-1630-c5* *Collinsella aerofaciens* *Eubacterium biforme* *Faecalibacterium prausnitzii* Prevotella copri** *Ruminococcus gnavus* *Unclassified* *Others*
**Group II** *Microecosystem*	Bacillaceae** Bacteroidaceae Christensenellaceae Clostridiaceae Erysipelotrichaceae Lachnospiraceae Lactobacillaceae** Mogibacteriaceae Paraprevotellaceae*** Pirellulaceae Porphyromonodaceae Prevotellaceae*** RFP12 **Ruminococcaceae** **S24-7** Spirochaetaceae** Streptococcaceae* Veillonellaceae Verrucomicrobiaceae p-2534-18B5 Unclassified Others	02d06 Akkermansia Bacillus Bacteroides Lactobacillus **Oscillospira** Parabacteroides Prevotella Ruminococcus Streptococcus Treponema p75-a5 **Unclassified** Others	*Bacillus cereus* *Eubacterium biforme* *Lactobacillus reuteri* Prevotella copri** Prevotella stercorea*** *Unclassified* *Others*
**Group III** *Diarrhea*	Bacteroidaceae Desulfovibrionaceae Enterobacteriaceae Erysipelotrichaceae Fusobacteriaceae Helicobacteriaceae Lachnospiraceae Mogibacteriaceae **Odoribacteraceae ^** Paraprevotellaceae*** Porphyromonodaceae Prevotellaceae Rikenellaceae Ruminococcaceae S24-7 Veillonellaceae Unclassified Others	Bacteroides Bilophila Blautia Butyricimonas Desulfovibrio Dorea Escherichia **Fusobacterium** **Odoribacter** Oscillospira Parabacteroides Phascolarctobacterium **Prevotella** Roseburia Ruminococcus Unclassified Others	***Escherichia coli ***^ *Faecalibacterium prausnitzii* *Parabacteroides distasonis* ^ Prevotella stercorea*** *Ruminococcus gnavus* *Unclassified* *Others*
**Group IV** *Antibiotics*	Bacillaceae Bacteroidaceae Coriobacteriaceae Desulfovibrionaceae Enterobacteriaceae Erysipelotrichaceae Fusobacteriaceae Helicobacteriaceae Lachnospiraceae Lactobacillaceae Odoribacteraceae ^ Paraprevotellaceae*** Porphyromonodaceae **Prevotellaceae** Rikenellaceae Ruminococcaceae **S24-7** Spirochaetaceae** Streptococcaceae* Veillonellaceae Unclassified Others	Bacteroides Bifidobacterium Blautia Bulleidia Dorea Escherichia Eubacterium Lactobacillus Megasphaera Oribacterium Oscillospira Parabacteroides Phascolarctobacterium Porphyromonas **Prevotella** Roseburia Sharpea Streptococcus Treponoma p-75-a5 Unclassified Others	*Bacillus cereus* ***Escherichia coli ***^ *Eubacterium biforme* *Parabacteroides distasonis* ^ Prevotella copri** Prevotella stercorea*** *Scharpea azabuensis* *Unclassified* *Others*

The most abundant bacteria in the sample or group of piglets are shown in bold. Single asterisk (*) shows bacterial families increased in both antibiotics and microecosystem groups. Double asterisk (**) shows family and species of bacteria present in healthy piglets and increased in both antibiotics and microecosystem groups. Triple asterisk (***) shows family and species of bacteria present in ill-diarrhea piglets but decreased in both antibiotics and microecosystem groups. ^ shows bacteria characteristic of diarrhea that remain in piglet gut flora after antibiotic treatment.

However, one major striking finding of our study in four groups of piglets in relation with infectious diarrhea was the differential regulation of the gut flora after consumption of antibiotics versus a more natural microecosystem treatment. Different preparations of *L. plantarum* have been shown to induce differential immune nuclear factor pathways in the duodenum of human and mice, supporting the idea that a specific microbial treatment aims at stimulating the immune system to establish more healthy conditions^52–54^. We show here that a preparation of *B. subtilis* (Y-15), *B. amyloliquefaciens* (DN6502) and *B. licheniformis* (SDZD02) is extremely efficient in curing piglet diarrhea via a beneficial effect on the gut (Figs. 3, 4, 5; Tables 3, 4). After treatment with our preparation of three bacilli cocktail (i.e. Microecosystem), the composition of the gut flora is very similar to that observed in healthy control conditions, much more than the gut flora of animals treated with antibiotics (see Figs. 2, 3, 4, 5; Figures S1–S5).

Healthy control piglets (Group I) and ill piglets treated by Microecosystem (Group II) show a very similar profile of bacteria at the phylum, class, order, family, genus and species level. The two groups of piglets (healthy and microecosystem) are both characterized by high levels of Firmicutes, Clostridia, Clostridiales, *Ruminococcaceae and* *S24-7*. The microbiome is also kept diverse in the two groups, healthy and microecosystem. Overall, the detail composition of the gut microbiota clearly shows shared patterns of gut microbiome in healthy and microecosystem groups. Bacteroidetes, Firmicutes, Proteobacteria, Spirochaetes, Verrucomicrobia (Phylum), Bacilli, Bacteroidia, Clostridia, Erysipelotrichia, Mollicutes, Planctomycetia, Spirochaetes, Verruchomicrobiae (Class), Bacillales, Bacteroidales, Clostridiales, Erysipelotrichales, Lactobacillales, Pirellulales, RF39, Spirocheatales (Order), *Bacillaceae, Christensenellaceae, Clostridiaceae, Erysipelotrichaceae, Lachnospiraceae, Lactobacillaceae, Mogibacteriaceae, Paraprevotellaceae, Pirellulaceae, Porphyromonadaceae, Prevotellaceae, RFP12, Ruminococcaceae, S24-7, Spirochaetaceae, Veillonellaceae* (Family), *02d06, Bacillus, Bacteroides, Lactobacillus, Oscillospira, Parabacteroides, Prevotella, Ruminococcus, Streptococcus, Treponema, p75-a5* (Genus), *Bacillus cereus*, *Eubacterium biforme* and *Prevotella copri* (Species), and Firmicutes-Clostridia-Clostridiales-*Ruminococcaceae*-*S24-7* was the most prevalent group of bacteria in both healthy control and microecosystem-treated piglets (Tables 3, 4).

In summary, microbiomes of healthy piglets and ill piglets treated with bacilli have in common five phyla, eight classes, eight orders, sixteen families, eleven genera and three species of bacteria. The only difference is found in the composition of the gut flora at the level of Genus. High levels of *Bifidobacterium* are found in Group I of healthy piglets (see Figs. 3, 4, 5; Tables 3, 4). No *Bifidobacterium* is induced after treatment with Bacilli, but microecosystem induced a peak of *Oscillospira*, which seems to be essential for the recovery of health in piglets affected by infectious diarrhea (see Figs. 3, 4, 5; Tables 3, 4). *Oscillospira* is a commensal gut bacterial genus that is extremely useful to digest resistant starches and ferments them in the intestine or rumen of mammals^55^. This bacterial genus has never been cultured but is known to be positively associated with leanness and health in human^56^. Therefore, it becomes an important challenge to succeed in producing cultured strains of *Oscillospira*, not only for piglet and cattle health, but also for human health.

In addition to revealing the composition of the microbiome positively associated with health in industrial piglets, we demonstrate that antibiotics (C_52_H_98_N_16_O_13_ and C_40_H_67_NO_14_) is a totally inefficient system to attempt to reestablish a gut flora compatible with long-term healthy conditions in pig or swine. While healthy conditions and conditions after bacilli-treatment clearly have overlapping features, we demonstrate in piglets that conditions after antibiotics-treatments have a lot of overlapping features with ill (diarrhea)-conditions: Bacteroidetes, Firmicutes, Proteobacteria (Phylum), Bacteroidia, Betaproteobacteria, Clostridia, Deltaproteobacteria, Fusobacteria (Class), Bacteroidales, Campylobacterales, Clostridiales, Desulfovibrionales, Enterobacteriales, Erysipelotrichales, Fusobacteriales (Order), *Bacteriodaceae*, *Desulfovibrionaceae*, *Enterobacteriaceae*, *Erysipelotrichaceae*, *Fusobacteriaceae*, *Helicobacteriaceae*, *Lachnospiraceae*, *Odoribacteraceae*, *Paraprevotellaceae*, *Porphyromonadaceae*, *Prevotellaceae*, *Rikenellaceae*, *Ruminococcaceae*, *S24-7*, *Veillonellaceae* (Family), *Bacteroides*, *Blautia*, *Dorea*, *Escherichia*, *Oscillospira*, *Parabacteroides*, *Phascolarctobacterium*, *Prevotella*, *Roseburia* (Genus), *Escherichia coli*, *Parabacteroides distasonis* and *Prevotella stercorea* (Species), with low levels of Firmicutes-Clostridia-Clostridiales-*Ruminococcaceae*-*S24-7*, but extremely high levels of Bacteroidetes-Bacteroidia-Bacteroidales-*Prevotella* and *E. coli* found in both ill- and + AB-conditions (Groups III and IV; see Tables 3, 4). So, the microbiomes identified in group III (illness) and group IV (antibiotics) share three phyla, five classes, seven orders, fourteen families, nine genera and three species of bacteria, demonstrating that the treatment with antibiotics does not help restore a healthy gut flora, but rather helps maintain a gut flora very close to ill-conditions in industrial piglets (see Figs. 3, 4, 5, Figures S1–S5; Tables 3, 4).

Illness and infectious diarrhea in piglets is found to be associated with low firmicutes/high bacteroidetes as found for other diseases such as obesity and other metabolic disorders, as well as gastrointestinal malfunctions of inflammatory bowel diseases, i.e. ulcerative colitis and Crohn’s disease, and even neurological symptoms associated with disorders such as autism^57–60^. To lower firmicutes and to raise bacteroidetes in order to balance the ratio to normal healthy conditions, we find that antibiotics such as colistin and kitasamycin are not suitable (see Figs. 3, 4, 5, Figures S1–S5; Tables 3, 4). They are suitable for increase in levels of some beneficial bacterial genera such as *Bacillaceae*, *Coriobacteriaceae*, *Lactobacillaceae*, *Prevotellaceae*, *S24-7*, *Spirochaetaceae* and *Streptococcaceae*, but they are unable to reduce or lower enough the levels of *Odoribacteraceae* and *Paraprevotellacellaceae*, which are predominant in ill piglets (Figs. 3, 4, 5, Figure S2; Tables 3, 4). This may be a key issue in prevention and curing piglet diarrhea disease because these two bacteroidetes bacteria are known to be rather harmful. They associate with autoimmune diseases (i.e. systemic sclerosis, Sjogren’s syndrome and anti-phospholipid syndrome) and helminth *Trichuris* colonization and infection, prelude to many various diseases such as dracunculiasis, loiasis, cysticercosis and echinococcosis, which can affect skin, liver, lungs, muscles, eyes and brain^61–63^. In male mice, increased abundance of *Paraprevotellaceae* occurs during the development of hepatocellular carcinoma^64^. Thus, our new finding that microecosystem-3 Bacilli (*B. subtilis* Y-15, *B. amyloliquefaciens* DN6502/*B. licheniformis* SDZD02) can eradicate 100% of toxic bacteria such as *Odoribacteraceae* and *Paraprevotellaceae *appears to be crucial not only for cattle and animal health, well-being linked to sustainable development of meat industry, but also for the preservation of human health (see Figs. 3, 4, 5, Figure S2; Tables 3, 4).

Moreover, antibiotics and microecosystem showed completely different effects at the Species-Level. Drug intake increased levels of *Sharpea azabuensis* and *Prevotella copri*, while microecosystem, not the drugs, was particularly efficient in increasing the levels of *Lactobacillus reuteri* (see Figs. 3, 4, 5; Tables 3, 4). Such bacterial species such as *L. reuteri* can colonize many different body sites, including the gastrointestinal tract, urinary tract, skin and breast milk. *L. reuteri* is known as extremely effective in stopping harmful strains of *E. coli* affecting their hosts and in ameliorating disease due to *E. coli* in a significant manner^65,66^. In our study, drug intake had no or little effects on the levels of *E. coli*, while microecosystem treatment (3 bacilli) removed *E. coli* from piglet digestive tract (see Figs. 3, 4, 5; Tables 3, 4). The high abundance of many kinds of *E. coli* is well known to cause diarrhea, urinary tract infection, respiratory illness and pneumonia, and many other illnesses in human^67^. We show here that *E. coli* is positively associated with piglet diarrhea (see Figs. 3, 4, 5; Tables 3, 4). So, our bacilli-based bioproduct is not only useful for controlling the ratio firmicute/bacteroidete, but also extremely efficient for putting an end to *E. coli* infection.

Interestingly, we show that the oral intake of microecosystem based on bacilli helps not only to discard *E. coli*, but also to reduce the levels of other bad harmful toxic bacteria such as *P. stercorea*. *E. coli* and *P. stercorea* are both found to be positively related to piglet diarrhea (see Figs. 3, 4, 5; Tables 3, 4). Both drug and probiotic are efficient in treating the *P. stercorea* infection, but only probiotic acts on *E. coli*, arguing for using our probiotic preparation instead a common drug chemical for piglet health.

Also interestingly, relative abundance of spirochaetes and mollicutes was found to be low in ill-conditions, but found to be significantly up-regulated to normal conditions after drug and bacillus treatments (see Fig. 4A; Tables 3, 4). Therefore, increase of Spirochaetes (and Mollicutes) could be associated with better health conditions in industrial piglets (see Fig. 4A,B; Tables 3, 4). Observed species value, chao1 value and ACE value can reflect the species richness of community. In our study of four groups of piglets in relation with diarrhea, the species richness community is found to be very similar between healthy and ecosystem groups, much more than between healthy and AB groups. The species richness community from antibiotics group largely overlaps with that from the ill group (Figs. 3, 4, 5, Figures S1–5; Tables 2, 3, 4), demonstrating that a bioproduct such as 3 Bacilli (*B. subtilis* Y-15/*B. amyloliquefaciens* DN6502/*B. licheniformis* SDZD02) boosts the gut microbiome, takes in beneficial strains, combs *E. coli* and other toxic bacteria out, maintains a “healthy microbiome” (microbiome observed in healthy conditions) and therefore represents a natural and extremely efficient alternative to the use of drugs and antibiotics in the treatment and/or prevention of piglet diarrhea disease.

To our knowledge, our study is a unique comprehensive and comparative approach that analyzes simultaneous piglet diarrhea syndrome and the composition of gut flora occurring after 1-week treatment with three bacilli species. The comparative analysis is two fold: (1) healthy vs unhealthy and (2) drug vs natural bioproduct, revealing the profiles of bacterial species associated with the development of piglet diarrhea and those more beneficial that could pave the way to even more probiotic cocktails. Here, a complete, systematic and thorough analysis of the complete microbiomes in groups of piglets in relation with epidemic infectious diarrhea reveals the key bacteria mediating the disease, but also the key beneficial bacteria mediating the curing and protecting effect of bacilli. The idea of applying a mixture of bacteria with different properties (immune cell stimulus, plant growth promoter and killer of bad bacterial strains) should be used for many other purposes in life and medical sciences. Our results in pigs certainly pave the way to future attempts in curing infectious diseases in other industrial animal species such as rabbit, chicken, duck, donkey, horse, cow, goat, lamb and mouton, in domestic animals, as well as in endangered animal species such as the musk deer or Panda that can develop poor health in inadequate captivity or park reserve environment^68,69^. Poor health conditions as consequences of an impaired or deficient digestive system is often associated with problems in animal reproduction. If the animals in reserves fail to conceive, they will go extinct. If animals from livestock fail to conceive or develop epidemic diseases, the output of farmers and farming industry may collapse and the country or the continent might suffer severe financial loss, while human health might be severely threatened. Hence, great care and new medication must be given to feeding in-reserve and farm animals to ensure healthy conditions, optimal reproduction and thereby sustainability in the life biodiversity and profitability in the food industry. One major finding in our study is that piglets diarrhea can be cured by a tritherapy of bacilli via many beneficial effects on the gut flora, as an alternative to the use of chemicals and pharmaceutical drugs such as colistin and kitasamycin. If our tritherapy for microbial infection can also be used to treat diarrhea and symptoms of viral infection needs to be investigated with caution. However, it is likely that tri-B also works on viral infections because the treatment has strong beneficial effects on the composition of the gut flora and therefore is a powerful way to boost and strengthen the immune system in a significant manner. The example of our probiotic formula (*B. subtilis* Y-15/*B. amyloliquefaciens* DN6502/*B. licheniformis* SDZD02), which is demonstrated to be so beneficial for health in the piglet models, may pave the way for a thorough and systematic analysis of the key components mediating the beneficial effect of the bacilli for improved health in humans. Further research and investigations should be undertaken to test the effects of this new probiotic (Y15-DN6502-SDZD02) in clinical studies involving natural treatments of metabolic diseases. This may bring our update on the use and investigation of probiotic bacteria from industrial animal farming, veterinary science and endangered species to efficient natural biomedicine in human populations. Here, the merit of our work in piglets is that the probiotic is for immediate protection by oral ingestion and feed-based passive immunization. Furthermore, we deliver a proof of commercial feasibility for immunization or improved immune system in piglets. The probiotic reported can be readily used for testing on other animal systems. To deliver a proof in the efficiency of additional commercially available probiotics, the formula Y15-DN6502-SDZD02 may be transformed or improved in a multicomponent cocktail, eventually adding some beneficial bacteria (Firmicutes, Clostridia, Clostridiales, *Ruminococcaceae*, *S24-7*, *Bifidobacterium*, *Oscillospira*, *B. cereus*, *E. biforme* and *P. copri*) identified as particularly curative in treatment of infectious diarrhea diseases in our study on piglets. Our study focusing on using a 3 Bacilli cocktail in response to piglet diarrhea and the identification of healthy piglet microbiome has certainly the merit to serve as a basis for the development of an even larger family of medicinal microbial bioproducts to be explored by the animal farming industry and traditional human medicine, as alternative to chemical drugs. This is particularly important since the demand for animal meat for human consumption is rising globally at an unprecedented rate with an essential problem to solve, i.e. the resistance developed by toxic bacteria in response to antibiotics^70^ and the failure of antibiotics in restoring a beneficial healthy viable gut flora (our study).

## Materials and methods

### Experimental farming and piglet diarrhea treatment

Piglets (Durac × Landrace × Yorkshire) for high throughput industrial meat production were reared in experimental farms located in YuZhou city (Henan Province). They were reared in nursery beds maintained at 24–31 °C, 32–67% humidity with constant daylight exposure. Piglet epidemic diarrhea caused by bacterial enterotoxigenic pathogens such as *Escherichia coli*, *Clostridium perfringens* and *Salmonella* is very common in industrial animal farming and usually treated with antibiotics in China. Accordingly, piglets that showed marked diarrhea were selected for experiments and kept in individual cages; each cage had one piglet to avoid ‘co-housing-impact on the pigs microbiome’. About 7.0–7.68 kg, 25 ± 1-days-old, and watery stool weaned piglets (half males and half females) were selected for treatment experiments. They were divided into four groups of twenty piglets each as described in Fig. 1. The piglets in the experimental group treated with antibiotics (AB, antibiotics group) were fed with a diet (maize germ, pollard, soya cake, fishmeal, lime, bonemeal, salt, lysine, feed premix, zinc and about 22% of Digestible Crude Protein) complemented with 400 ppm colistin (C_52_H_98_N_16_O_13_) and 100 ppm kitasamycin (C_40_H_67_NO_14_). The piglets in the experimental group treated with probiotics (microecosystem) were fed with a diet complemented with 3000 ppm natural products instead of antibiotics. The probiotic formula (“Direct fed microbial”) contained mixed *Bacillus subtilis* Y-15, *B. amyloliquefaciens* DN6502 and *B. licheniformsis* SDZD02 (3.0 × 10^10^ CFU/g; 1.0 × 10^10^ CFU/g of each strain), produced by Shandong Global Biotech Co. Ltd. The piglets in the healthy control group (normal) never showed any clinical manifestations of illness (no diarrhea). They displayed a normal behavior. They showed no behavioral feeding problems and maintained normal growth. They were fed with a diet complemented with 3000 ppm placebo (*Bacillus*-free carrier of probiotics product). Development of piglet diarrhea was diagnosed on the basis of the number of affected litters, the number of pigs affected by litter, and the age of pigs affected^71^. Watery diarrhea was not due to an experimental infection of newly born piglets but occurred naturally. All piglets had the same symptoms (watery diarrhea) and therefore the same bacterial infection. Piglets with clinical signs of diarrhea represented ill cases or conditions. The treatment was ongoing for 7 days. The incidence of diarrhea was measured and diarrhea went away after 7 days treatment in antibiotics and natural product (microecosystem) groups of piglets. After 7 days treatment, the therapeutic effects in both antibiotics and microecosystem groups reached significantly improved and very stable conditions. The follow up of the experiment was 1 month. No piglet diarrhea symptoms were observed for up to 1 month, in particular in the bacillus group. Fecal samples were collected only once per piglet in each group after 7 days treatment and stored at − 80 °C until DNA extraction, microbiome analysis and calculation of bacteria/diversity indices between the four groups (Fig. 1).

### Illumina library construction

Feces were collected in the litters of ten piglets in the four different groups after 7 days of treatment (Fig. 1). The fecal samples were placed in sterile tubes and stored at − 80 °C. To determine the bacterial composition from piglets in the four different groups, 2.0 g of fecal samples from Group I–IV pigs were processed for genomic DNA extraction using QIAamp Fast DNA Stool MiniKit (Qiagen GmbH, Hilden, Germany) according to the manufacturer’s and user’s instructions^72^.

Following Yue et al. (2014) on mice models^27^, total genomic DNA from piglets was used as template (10 ng) in PCR reactions employing 16F and 16S universal primers 16F 5′**-**CGC CCG GGG CGC GCC CCG GGCGGG GCG GGG GCA CGG GGG G AGA GTT TGA TCM TGG CTC AG-3′ and 16R 5′-TAC GGY TAC CTT GTT ACG ACT T-3′ (Invitrogen, Shanghai, China)^27^. PCR amplification of 16S rDNA products (TransGen Biotech, Beijing, China) was carried out in a Takara Master Thermal Cycler Dice (Takara, Dalian, China) programmed for an initial denaturation of 95 °C for 3 min followed by 30 cycles of (94 °C for 30 s), 50 °C for 30 s, 72 °C for 1 min and a final extension of 72 °C for 7 min^27^. PCR products for each group were purified using agarose gel electrophoresis (Bio-Rad Beijing, China) and QIAquick gel extraction kit (Qiagen Inc., Valencia, California, USA) so as to remove any residuals. The final DNA concentration for each sample was determined using a Thermo Scientific™ NanoDrop 2000 spectrophotometer (Thermo Fisher Scientific, UK) before sequencing at the Beijing Genomics Institute (BGI Co., Ltd).

### High-throughput sequencing of piglet fecal DNA

The 16S rDNA products from normal, diarrhea, antibiotics and microecosystem groups (see Fig. 1) were used for Illumina sequencing (BGI Co., Ltd). Raw sequence data were pre-processed to get clean data by in-house (BGI Co., Ltd) procedure as following. Truncation of sequence reads not having an average quality of 20 over a 30 bp sliding window based on the Phred quality score algorithm, and trimmed reads having less than 75% of their original length, as well as its paired read, were removed. Reads contaminated by adapter (default parameter: 15 bases overlapped by reads and adapter with maximal 3 bases mismatch allowed), reads with low complexity (default: reads with 10 consecutive same base) and reads with ambiguous basa (N base), and its paired reads, were also removed as also described in Lai et al. (2016) and Menghini et al. (2019)^73,74^.

For pooling library with barcode samples mixed, the clean reads were assigned to corresponding samples by allowing 0 base mismatch to barcode sequences with in-house scripts (BGI Co., Ltd)^73,74^. Paired-end reads were generated with Illumina HiSeq/MiSeq platform, then the reads with sequencing adapters, N base, poly base, low quality etc. were filtered out with default parameters (BGI Co., Ltd)^73,74^. Data processing results including Reads Length (RL), Raw Data (RD), Data Utilization Ratio (DUR) and Read Utilization Ratio (RUR) are reported in Table S1 (BGI Co., Ltd). Then paired end reads were merged to tags. If the two paired-end reads overlapped, the consensus sequence was generated by FLASH (Fast Length Adjustment of Short reads, v1.2.11), and the detailed method was as follows: (1) minimal overlapping length: 15 bp, 2) mismatching ratio of overlapped region: ≤ 0.1. Paired end reads without overlaps were removed. The high quality paired-end reads were combined to tags based on overlaps.

About 53,559 tags were obtained in total with 133,639 tags per sample on average, and the average is 252 bp (see Table S2; BGI Co., Ltd).

### Analysis of community patterns

The tags were clustered to OTU (Operational Taxonomic Unit) by scripts of software USEARCH (v7.0.1090). The tags were clustered into OTU with a 97% threshold by using UPARSE, and the OTU unique representative sequences were obtained. Chimeras were filtered out using UCHIME (v4.2.40). The 16S rDNA and ITS sequences were screened for chimeras by mapping to gold database (v20110519), UNITE (v20140703) separately, de novo chimera detection was done for 18S rDNA sequences. All tags were mapped to each OTU representative sequences using USEARCH GLOBAL (Table S3). OTU representative sequences were taxonomically classified using Ribosomal Database Project (RDP; Release9 201203)^75^ Classifier v.2.2 trained on the Greengenes database (V201305)^76^, using 0.8 confidence values as cutoff. Unassigned OTUs and OTUS not assigned to specific target species were removed. Filtered OTUs were used to final processing, i.e. OTU cluster and abundance as a measurement of diversity in bacterial communities from antibiotics, diarrhea, microecosystem and normal group samples (BGI Co., Ltd).

Venn diagram was used to visually display the number of common/unique OTUs in the 4-samples/groups. The core microbiome of the different groups was obtained when combined with the OTU representing species. Based on the OTU abundance, OTU of each group was listed, and Venn diagram was drawn by VennDiagram of software R(v3.1.1), then the common and specific OTU ID were summarized (see Fig. 2; BGI Co., Ltd).

In order to display the differences of OTU composition in between the four samples or groups, principal component analysis (PCA) was used to construct 2-D graph to summarize factors mainly responsible for this difference, similarly to the study of Liu et al. (2020)^77^. As for Liu et al. (2020)^77^, similarity is high if two samples or groups are closely related. Based on the OTU abundance information (see Table 1), the relative abundance of each OTU in each sample/group was calculated, and the PCA of OTU was done with the relative abundance value (Figure S1A)^77^. The software used in this step was package “ade4” of software R(v3.1.1)^78^. In our study, OTU rank curve was also used as a means for virtually representing species richness and species evenness among the four piglet samples or groups in relation with ill-diarrhea and curing (Figure S1B). For OTU rank curve, the package software was also R(v3.1.1)^78^. Then, the tags number of each taxonomic rank (phylum, class, order, family, genus, species) or OTU in different samples were summarized in a profiling table or histogram, and the histogram was drawn with the software R(v3.1.1) as also described in Liu et al. (2020)^77,78^. In our study, bacterial species clustering based on the abundance of each species in four piglet samples related to infectious diarrhea was shown by heatmap^79^. Species heat map analysis was done based on the relative abundance of each species in each sample^77–79^. To minimize the differences degree of the relative abundance value, the values were all log transformed. If the relative abundance of certain species is 0, the half of the minimum abundance value will be substituted for it. Accordingly, heatmaps were generated using the package “gplots” of software R(v3.1.1)^80^ and the distance algorithm is “euclidean”, the clustering method is “complete” as also used by Solanki et al. (2020)^81^ (BGI Co., Ltd).

### Phylogenetic analysis of microbial bacteria from piglet fecal samples

For phylogenetic analysis, the representative bacterial sequences were aligned against the Silva core set (Silva_core_alignment_seqs) using PyNAST by “align_seqs.py”^82^. A representative OTU phylogenetic tree was constructed using Quantitative Insights Into Microbial Ecology (QIIME, v1.80) built-in scripts including the fasttree method for tree construction^83^. The tags with highest abundance of each Genus were chosen as the corresponding Genus representative sequences, and Genus level phylogenetic tree was obtained by the same way of OTU phylogenetic tree as also described in Zhang et al. (2019)^84^. The phylogenetic tree was imaged by software R(v3.1.1) at last (BGI Co., Ltd)^85^.

### Analysis of species abundance, richness and diversity

Alpha diversity was applied for analyzing complexity of species diversity for the four samples through several indices, including observed species, chao 1, ace, Shannon and Simpson^86–88^. The complexity of sample is proportional with the first four values, while with a negative correlation with Simpson value. The rarefaction curve based on the three values (species value, chao1 value and ACE value) was used to evaluate if produced data was enough to cover all species in the community. When the curve tends to be smooth, it suggests that the produced data is enough. Otherwise, when the curve continues to climb with increasing sequencing effort, it shows a high complexity in samples, and there still be species uncovered by the sequencing data. The indices were calculated by Mothur(v1.31.2), and the corresponding rarefaction curve was drawn by software R(v3.1.1). The calculation formula of each index referred to https://www.mothur.org/wiki/Calculators and the method of drawing rarefaction curve is as follows: (1) calculating OTU numbers based on extracted tags (in multiples of 500), and (2) rarefaction curve was drawn using the indices calculated with extracted tags. The samples belonged to four different groups, and the sample number in per group was more than three, so further differential analysis among group was done using the alpha diversity indices^89^. Wilcoxon Rank-Sum Test was used for two groups comparison^90^, while Kruskal–Wallis Test was used for multi-groups comparison^91^. Plotbox of alpha-diversity was drawn, and complete alpha diversity statistics was done by software R(v3.1.1) (see Table 2; BGI Co., Ltd).

### Animal (pig) experimental study statements

(i) We confirm that all methods were carried out in accordance with relevant guidelines and regulations.
(ii) We confirm that all experimental protocols were approved by a named institutional and/or licensing committee/s.
(iii) We confirm that the use of live animals (piglets) in this study was approved by the Shandong University Animal Research Ethics Committee and was licensed by Shandong Province (Governmental license SCXK Lu 20090001). We also confirm that all experimental protocols used in our study, animal care and use, were approved by the Institutional Ethics Committee of HAU and Henan Province (Permit number 11-0085).

## Supplementary information

Supplementary Figure S1.

Supplementary Figure S2.

Supplementary Figure S3.

Supplementary Figure S4.

Supplementary Figure S5.

Supplementary Table S1.

Supplementary Table S2.

Supplementary Table S3.

Supplementary Captions.
