# Supplementary material for: Curing piglets from diarrhea and preparation of a healthy microbiome with Bacillus treatment for industrial animal breeding
Source: Sci Rep. 2020 Nov 10;10:19476. doi: 10.1038/s41598-020-75207-1 (PMC7656456; doi:10.1038/s41598-020-75207-1)
Supplement: Supplementary file 1 — Supplementary Figure S1. [file 41598_2020_75207_MOESM1_ESM.pdf]

"Curing piglets from diarrhea and preparation of a healthy microbiome with Bacillus treatment for industrial animal breeding"

Shousong Yue, Zhentian Li, Fuli Hu, and Jean-François Picimbon

Figure S1

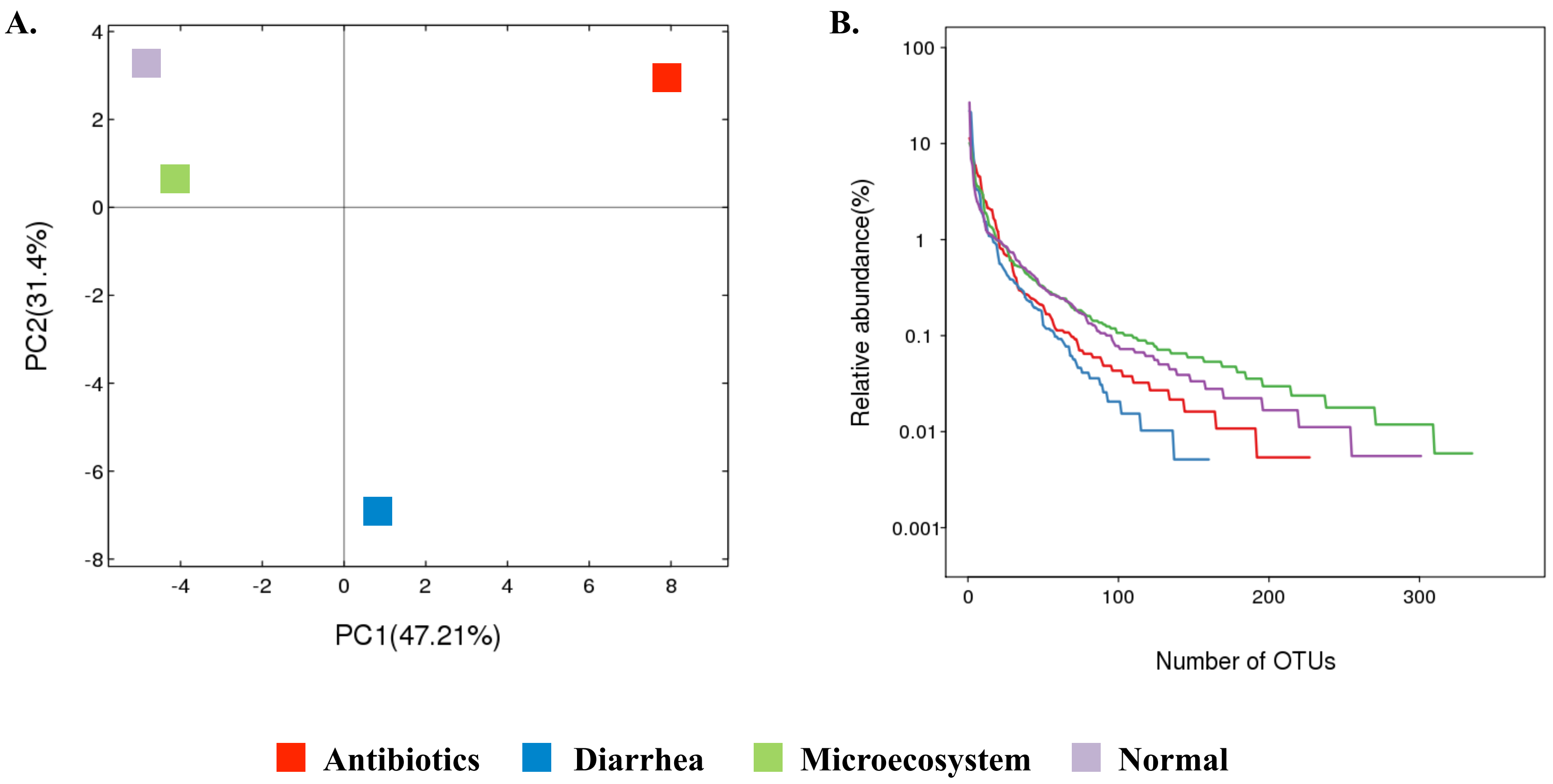
A.
PC2(31.4%)
PC1(47.21%)
4
2
0
-2
-4
-6
-8
-4
-2
0
2
4
6
8
B.
Relative abundance(%)
Number of OTUs
100
10
1
0.1
0.01
0.001
0
100
200
300
Antibiotics
Diarrhea
Microecosystem
Normal
