# Supplementary figures and images for: Curing piglets from diarrhea and preparation of a healthy microbiome with Bacillus treatment for industrial animal breeding
Source: Sci Rep. 2020 Nov 10;10:19476. doi: 10.1038/s41598-020-75207-1 (PMC7656456; doi:10.1038/s41598-020-75207-1)

**A.**

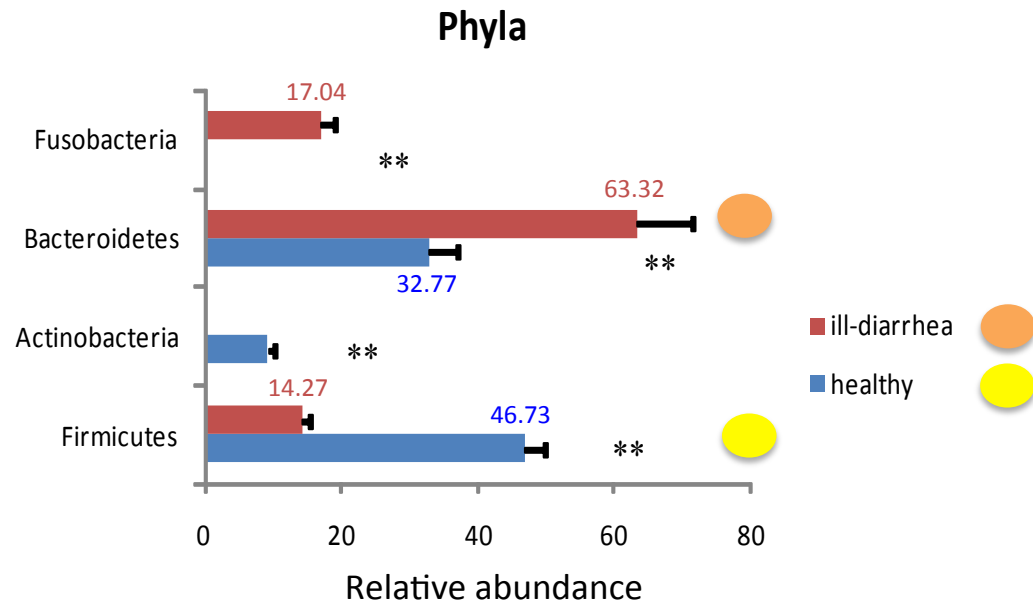

**B.**

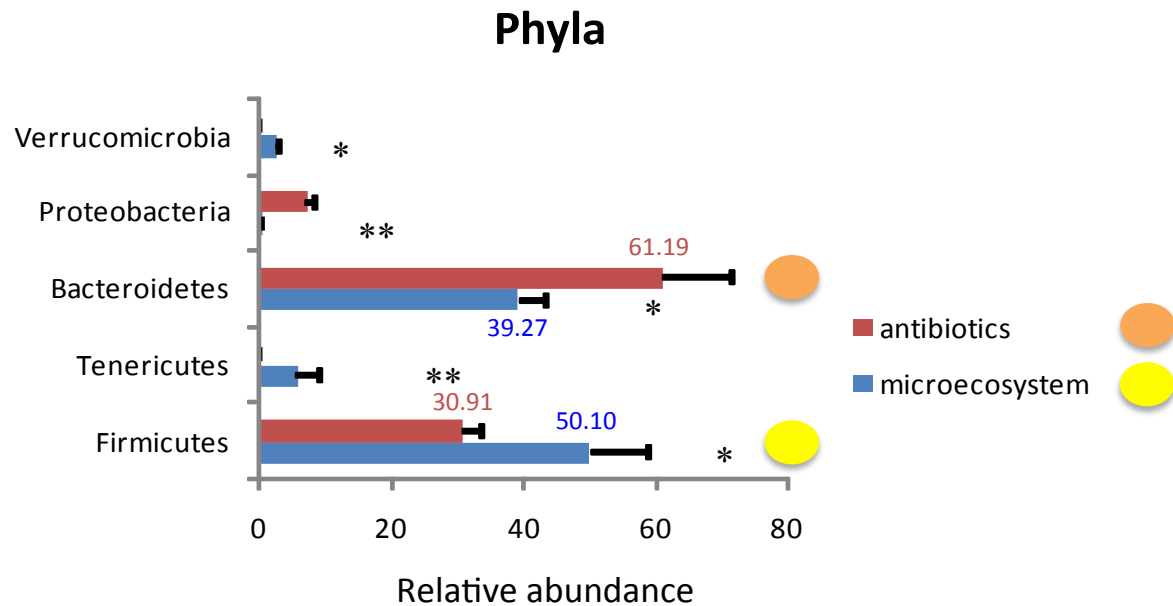

Supplement: Supplementary file 2 — Supplementary Figure S2. [file 41598_2020_75207_MOESM2_ESM.pdf]

Antibiotics Diarrhea Microecosystem Normal

A.

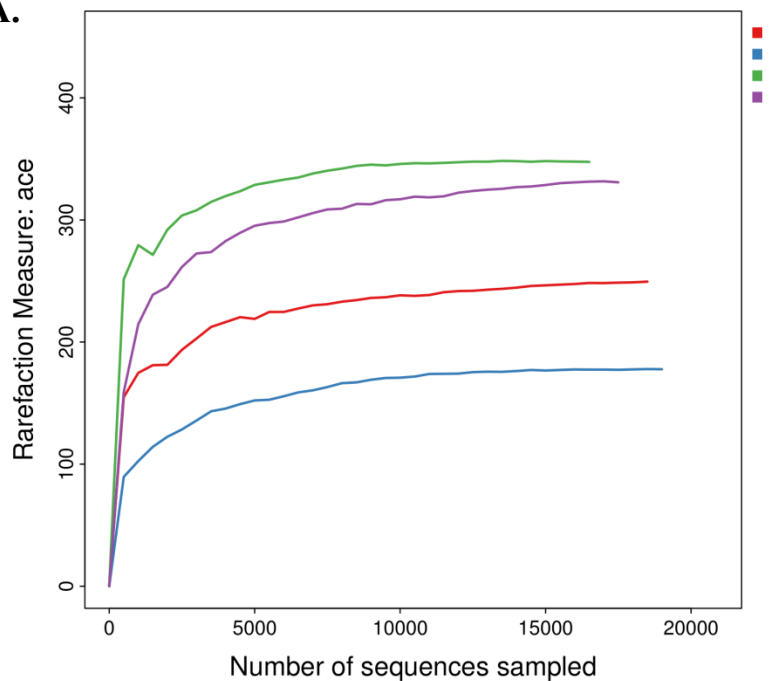

B.

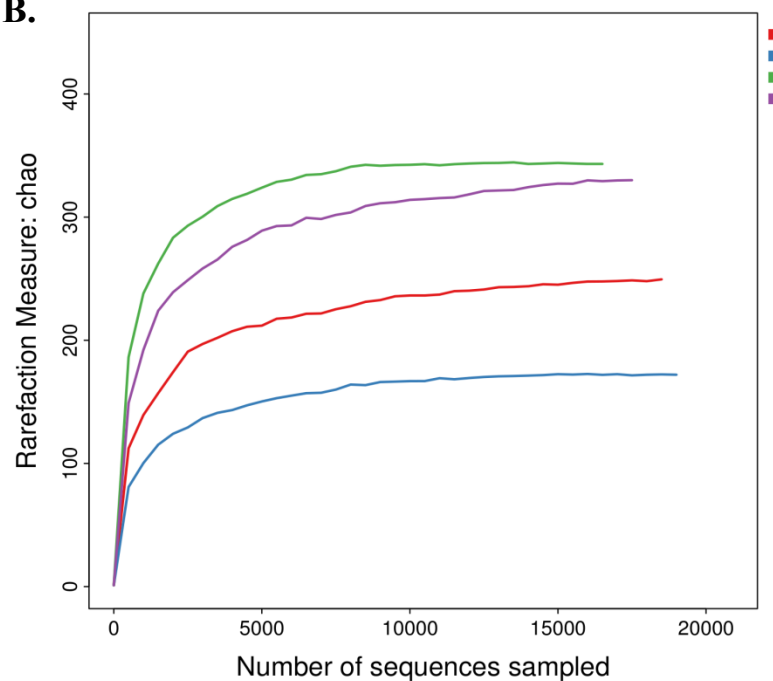

C.

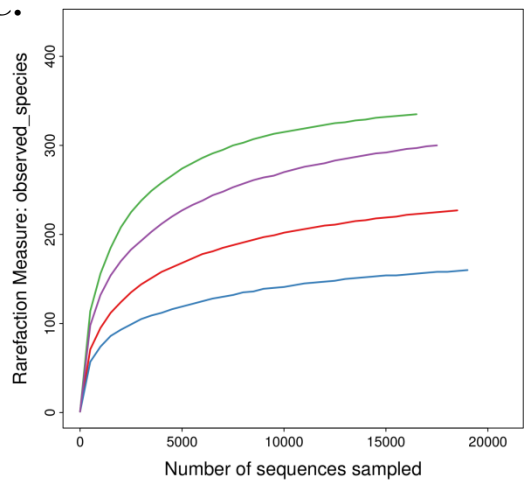

D.

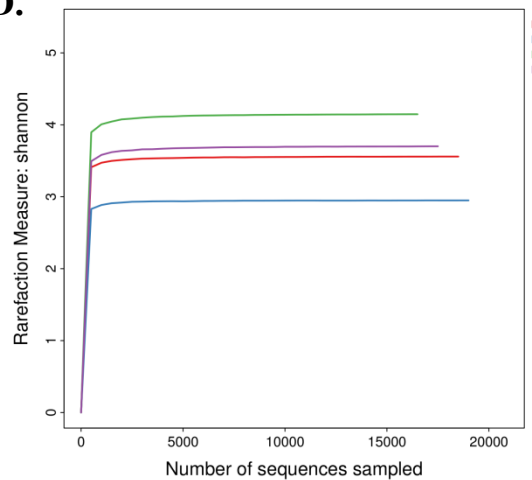

E.

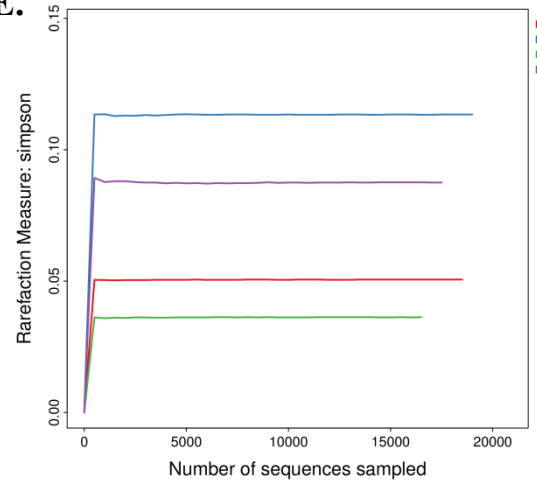

Supplement: Supplementary file 3 — Supplementary Figure S3. [file 41598_2020_75207_MOESM3_ESM.pdf]

**A**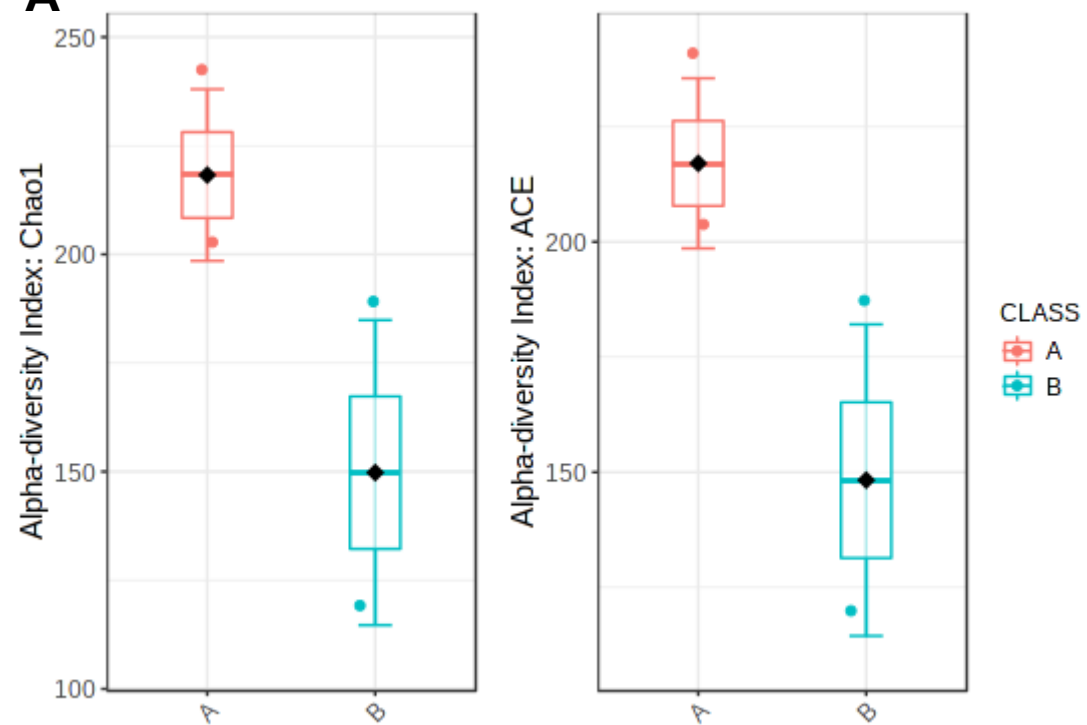**B**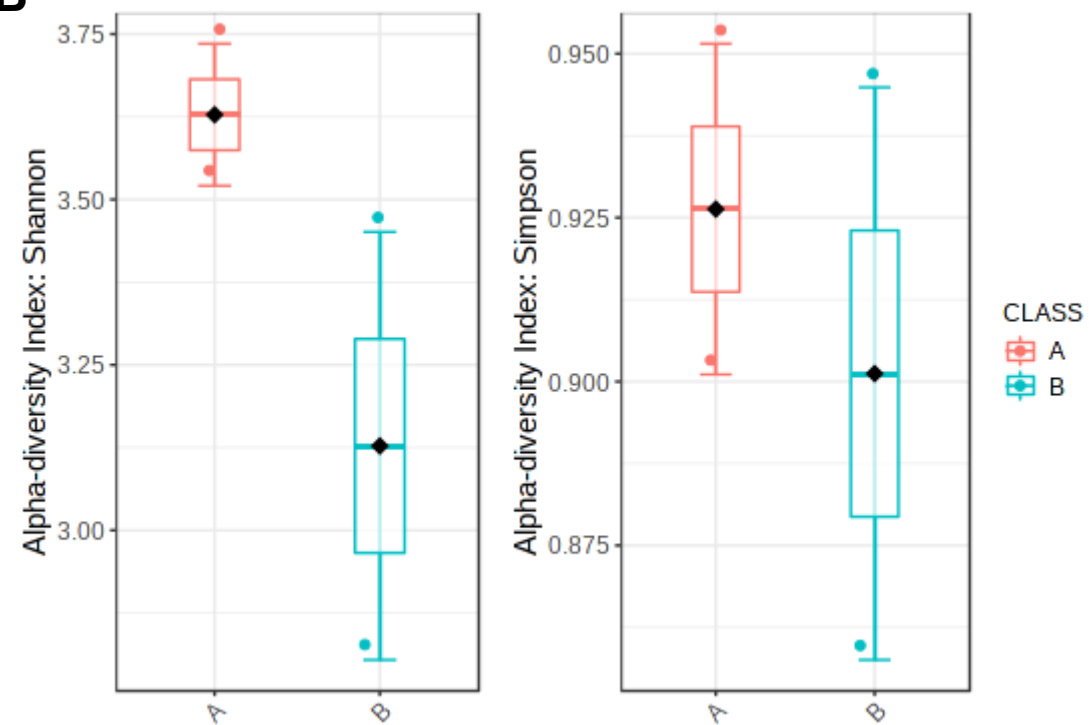

Supplement: Supplementary file 4 — Supplementary Figure S4. [file 41598_2020_75207_MOESM4_ESM.pdf]

**A**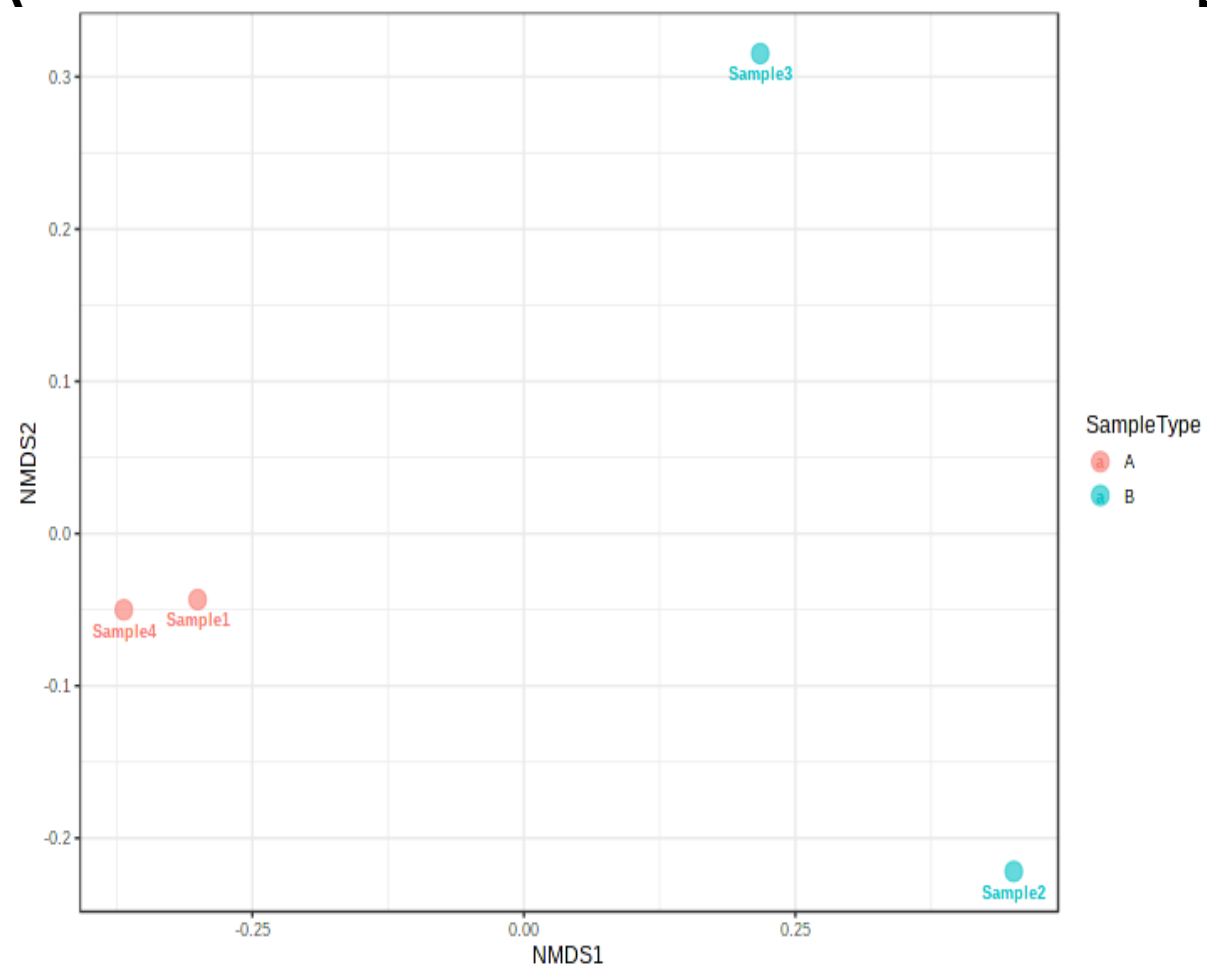**B**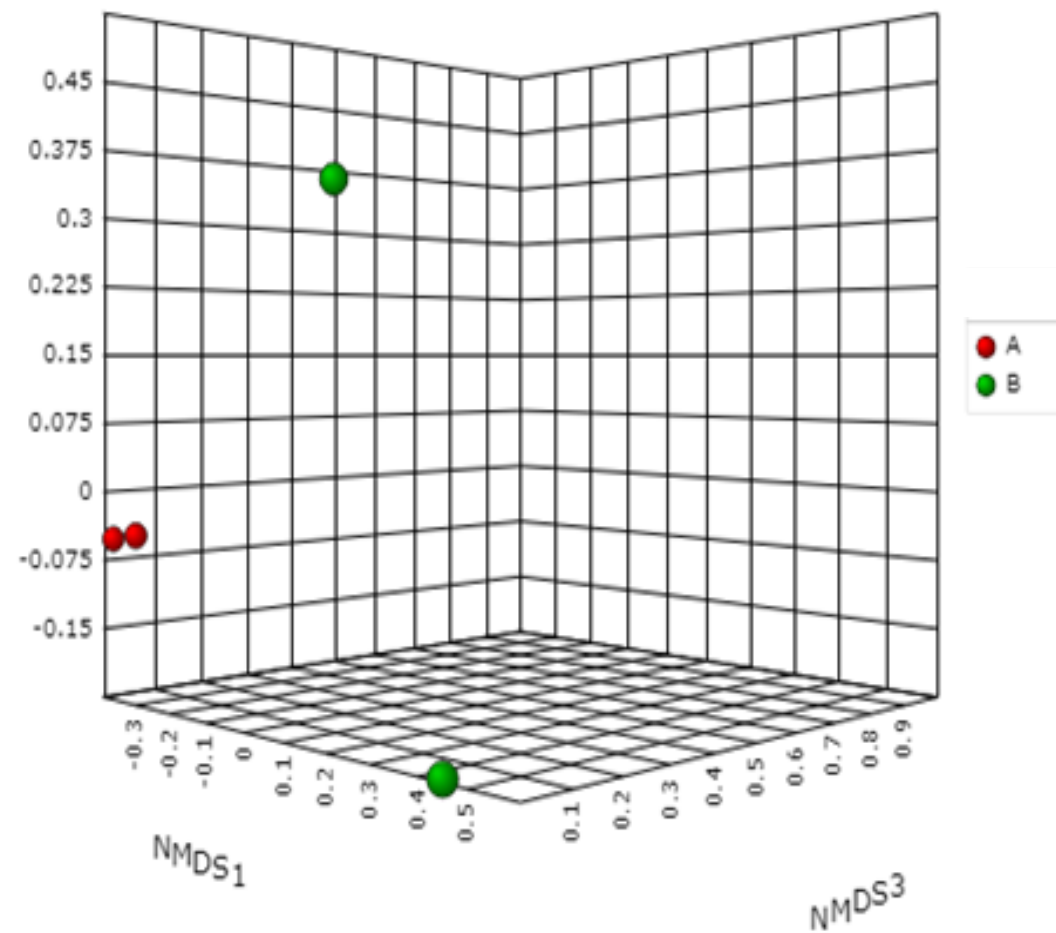

Supplement: Supplementary file 5 — Supplementary Figure S5. [file 41598_2020_75207_MOESM5_ESM.pdf]
