# Supplementary material for: Curing piglets from diarrhea and preparation of a healthy microbiome with Bacillus treatment for industrial animal breeding
Source: Sci Rep. 2020 Nov 10;10:19476. doi: 10.1038/s41598-020-75207-1 (PMC7656456; doi:10.1038/s41598-020-75207-1)
Supplement: Supplementary file 6 — Supplementary Table S1. [file 41598_2020_75207_MOESM6_ESM.docx]

| **Sample** | **RL**  **(bp)** | **RD**  **(mbp)** | **Adapter**  **(%)** | **N base**  **(%)** | **Ploy base**  **(%)** | **Low Quality**  **(%)** | **Clean Data**  **(Mbp)** | **DUR**  **(%)** | **Raw Reads** | **Clean Reads** | **RUR**  **(%)** |
| --- | --- | --- | --- | --- | --- | --- | --- | --- | --- | --- | --- |
| *Antibiotics* | 250:250 | 77.86 | 0.000 | 0.000 | 0.014 | 8.370 | 65.40 | 84.00 | 155729*2 | 131266*2 | 84.29 |
| *Diarrhea* | 250:250 | 79.89 | 0.000 | 0.000 | 0.011 | 4.198 | 73.62 | 92.16 | 147708*2 | 147708*2 | 92.45 |
| *Microecosystem* | 250:250 | 70.41 | 0.000 | 0.000 | 0.027 | 4.088 | 65.24 | 92.65 | 140822*2 | 130787*2 | 92.85 |
| *Normal* | 250:250 | 67.93 | 0.000 | 0.000 | 0.017 | 3.258 | 64.14 | 94.43 | 135853*2 | 128548*2 | 94.62 |

**Table S1.** Data processing results. RL: Reads Length, RD: Raw Data, DUR: Data Utilization Ratio, RUR: Read Utilization Ratio.

"Curing piglets from diarrhea and preparation of a healthy microbiome with Bacillus treatment for industrial animal breeding"

Shousong Yue, Zhentian Li, Fuli Hu, and Jean-François Picimbon
