# Supplementary material for: Curing piglets from diarrhea and preparation of a healthy microbiome with Bacillus treatment for industrial animal breeding
Source: Sci Rep. 2020 Nov 10;10:19476. doi: 10.1038/s41598-020-75207-1 (PMC7656456; doi:10.1038/s41598-020-75207-1)
Supplement: Supplementary file 7 — Supplementary Table S2. [file 41598_2020_75207_MOESM7_ESM.docx]

| **Sample** | **Total Pairs Read Number** | **Connect Tag Number** | **Connect Ratio (%)** | **Average Length and SD** | **Tags Without Primer** | **Tag UR**  **(%)** | **Average Length (bp)**  **and SD** |
| --- | --- | --- | --- | --- | --- | --- | --- |
| *Antibiotics* | 131266 | 129939 | 98.99 | 252/0 | - | - | -/- |
| *Diarrhea* | 147708 | 146750 | 99.35 | 252/0 | - | - | -/- |
| *Microecosystem* | 130787 | 129910 | 99.33 | 252/0 | - | - | -/- |
| *Normal* | 128548 | 127960 | 99.54 | 252/0 | - | - | -/- |

**Table S2.** Tags statistics. “-“ in Tags Without Primer” indicates there is no primer removal to the tags.

"Curing piglets from diarrhea and preparation of a healthy microbiome with Bacillus treatment for industrial animal breeding"

Shousong Yue, Zhentian Li, Fuli Hu, and Jean-François Picimbon
