# Supplementary material for: Curing piglets from diarrhea and preparation of a healthy microbiome with Bacillus treatment for industrial animal breeding
Source: Sci Rep. 2020 Nov 10;10:19476. doi: 10.1038/s41598-020-75207-1 (PMC7656456; doi:10.1038/s41598-020-75207-1)
Supplement: Supplementary file 8 — Supplementary Table S3. [file 41598_2020_75207_MOESM8_ESM.docx]

| **Sample** | **Tag Number** | **OTU Number** |
| --- | --- | --- |
| *Antibiotics* | 18549 | 227 |
| *Diarrhea* | 19477 | 160 |
| *Microecosystem* | 16848 | 335 |
| *Normal* | 17942 | 301 |

**Table S3.** Operational Taxonomic Unit (OTU) statistics (97% threshold). OTU number per sample primarily represents the degree of sample diversity

"Curing piglets from diarrhea and preparation of a healthy microbiome with Bacillus treatment for industrial animal breeding"

Shousong Yue, Zhentian Li, Fuli Hu, and Jean-François Picimbon
