# Supplementary material for: Curing piglets from diarrhea and preparation of a healthy microbiome with Bacillus treatment for industrial animal breeding
Source: Sci Rep. 2020 Nov 10;10:19476. doi: 10.1038/s41598-020-75207-1 (PMC7656456; doi:10.1038/s41598-020-75207-1)
Supplement: Supplementary file 9 — Supplementary Captions. [file 41598_2020_75207_MOESM9_ESM.docx]

**Fig. S1.** Differences of OTU composition in four groups of piglets in relation with ill-diarrhea and curing. A) Principal component analysis based on OTU abundance. X-axis, 1^st^ principal component; Y-axis, 2^nd^ principal component. Number in brackets represents contributions of principal components to differences among samples. A dot represents each sample, and different colors represent different groups. B) OTU rank curve. Species richness is viewed as the number of different species on the chart (X-axis). Species evenness is derived from the slope of the line that fits the graph. A steep gradient indicates low evenness as the high ranking species have much higher abundance than the flow ranking species. A shallow gradient indicates high evenness as the abundances of different species are similar (ade4, R(v3.1.1), BGI Co., Ltd).

**Fig. S2** Main bacterial phyla identified in piglet samples related to ill-diarrhea and curing. A) Comparative analysis between ill-diarrhea and healthy control conditions. B) Comparative analysis between antibiotics and microecosystem treatments. The circle in orange shows main bacterial phyla in ill-diarrhea conditions and antibiotics treatments. The circle in yellow shows main bacterial phyla in healthy conditions and microecosystem treatments (*: p < 0.05, **: p < 0.01; Wilcoxon Rank-Sum Test).

**Fig. S3** The different curves based on observed species, chao1, ace, shannon and simpson values (Mothur(v1.31.2), R(v3.1.1), BGI Co., Ltd).

**Fig. S4** Alpha-diversity analysis of grouped samples. A) Chao1 and ACE indices. B) Shannon and Simpson values (MicrobiomeAnalyst). Class A: healthy normal and microecosystem, Class B: Ill and antibiotics.

**Fig. S5** Beta-diversity analysis of grouped samples. Nonmetric multidimensional scaling (NMDS) ordination analysis with Bray–Curtis distance showing that the gut microbiota of healthy normal (Sample 1) and microecosystem (Sample 4) group together separately from ill-diarrhea (Sample 2) and antibiotics (Sample 3). A) NMDS- 2D plot. Sample distribution: healthy normal and microecosystem (pink dots), ill-diarrhea and antibiotics (blue dots). B) NMDS-3D plot. Sample distribution: healthy normal and microecosystem (red dots), ill-diarrhea and antibiotics (green dots). Permutational multivariate analysis of variance (PERMANOVA) by adonis in the vegan R package.
